# Combining Subjective Perceptions and Objective Behavioral Metrics With the Elderly Digital Twin System: Quantitative Usability Study

**DOI:** 10.2196/91873

**Published:** 2026-05-26

**Authors:** Ziaullah Momand, Pornchai Mongkolnam, Debajyoti Pal, Siam Yamsaengsung

**Affiliations:** 1 School of Information Technology, King Mongkut's University of Technology Thonburi, 126 Pracha Uthit Rd, Bang Mod, Thung Khru, Bangkok, 10140, Thailand, 66 24709892

**Keywords:** elderly digital twin, usability study, System Usability Scale, user engagement score, caregiver support

## Abstract

**Background:**

The growing aging population has increased the need for technologies that support informal caregivers in home-based older adult care. Digital twin (DT) systems offer promising capabilities; yet, their effectiveness depends on usability, an aspect still insufficiently evaluated among caregivers.

**Objective:**

This study aimed to assess the usability of an older adult care DT system using a dual-method evaluation that integrates subjective and objective behavioral performance.

**Methods:**

Fifty caregivers participated in a usability assessment combining the System Usability Scale (SUS) and detailed system activity log analytics. Log-based measures included task completion, time on task, errors, and abandonment rate. A composite user engagement score was computed and analyzed for correlation and predictive association with SUS ratings. Engagement clusters were also explored.

**Results:**

Caregivers reported an excellent mean SUS score of 80.45. System logs showed a 94.08% task completion rate, 2.66% abandonment, and an average task duration of 89.16 seconds. User engagement score demonstrated significant correlations with SUS (*r*=0.626, ρ=0.552; *P*<.001) and significantly predicted usability in regression analysis (*β*=52.94, *R*²=0.392; *P*<.001). Engagement-based clustering identified high-, medium-, and low-tier user groups, each exhibiting distinct usability patterns.

**Conclusions:**

Integrating subjective usability ratings with objective behavioral metrics provides a rigorous and comprehensive approach to evaluating DT systems for older adult care. The findings highlight strong usability of the system and offer actionable insights for refining caregiver support technologies.

## Introduction

### Background

The global demographic shift toward an aging population presents unprecedented challenges for health care systems worldwide. The world’s population older than 60 years is projected to double from 1.06 billion (13.5%) in 2020 to 2.13 billion in 2050 (22.0%), raising significant concerns about the impact of aging [1]. This rapid aging process is characterized by an increased prevalence of chronic diseases, complex health care needs, and a growing demand for personalized and proactive health care models [2,3]. In the current technology-driven age, digital twin (DT) has emerged as a transformative approach in health care, offering the potential to revolutionize older adult care through predictive analytics and personalized interventions. DTs are virtual representations of physical entities that enable the dynamic simulation of potential treatment strategies, monitoring and prediction of health trajectories, and early intervention and prevention [4]. In the context of older adult health monitoring, DTs create comprehensive virtual models that integrate multimodal data, including clinical records, genetic information, wearable sensor data, and environmental factors, to provide a holistic view of an individual’s health status [5,6].

The application of DT technology in older adult care is particularly compelling because of its ability to monitor vital signs, physiological parameters, and other health-related data in real time, enabling health care providers to detect early signs of deterioration or anomalies and proactively intervene in such scenarios [6]. Recent implementations have demonstrated the potential of this technology in aging-in-place scenarios, where DTs facilitate continuous health monitoring while preserving the independence and quality of life of seniors [7]. For example, DT systems have been successfully deployed to monitor older adult patients for fall detection, abnormal posture recognition, and health risk assessment, with validation trials showing promising results in real-world nursing home environments [8].

The complexity of older adult care often necessitates significant involvement from both formal and informal caregivers, who play crucial roles in health monitoring, decision-making, and care coordination. Informal caregivers, predominantly family members, face substantial challenges in navigating complex health care systems and making informed decisions [9,10]. Family caregivers’ decision support interventions have shown promise in improving care outcomes, with studies demonstrating that well-designed decision support tools can reduce family caregivers’ decision uncertainty and improve satisfaction with the quality of care [9]. Such tools leverage trust, cultural humility, strength-based approaches, and effective information sharing to facilitate meaningful conversations between caregivers and health care providers. In the context of DT systems for older adult care, caregiver-facing interfaces have become essential components that must translate complex health data into actionable insights that support informed care–related decisions [10].

The success of DT systems in older adult health care depends critically on their usability, particularly in home care settings, where family members are involved in a variety of experiences [11]. In health care contexts, poor usability can have serious consequences, including medical errors, reduced adoption rates, and compromised patient safety [12]. For DT systems targeting older adult populations and their caregivers, usability considerations have become even more critical because of the potential age-related changes in vision, dexterity, and cognitive processing. Older adults often require caregiver mediation when interacting with digital health technologies, and systems must be designed to accommodate both direct older adult users and caregiver interactions [13].

In health care usability research, evaluation has largely relied on subjective self-report instruments, most prominently the System Usability Scale (SUS), which has been applied across diverse digital health systems such as caregiver monitoring tools HELMA [14], self-management apps for heart failure (Engage) [13], exergame training systems [15], and interdisciplinary hospital information platforms such as therapy and monitoring systems [16]. While SUS provides standardized benchmarking, its reliance on user perception limits insights into actual system interaction. Studies have attempted to complement the SUS with performance or log data: the HELMA project tracked caregiver login frequency and session duration, the Engage system analyzed task completion and observed errors, and exergame interventions logged exercise scores and reaction accuracy. However, these measures often remain surface-level, focusing on frequency, duration, or clinical outcomes rather than granular task behavior analytics such as navigation sequences, error recovery, feature usage, task completion, time on task, click on task ratio, retry rate, session duration, or task abandonment ratio. Moreover, limitations such as older adults’ reliance on caregiver mediation [14,17] and testing in controlled settings [13,15] constrain the validity of existing evaluations. Collectively, these findings underscore a critical gap: while subjective usability assessments dominate and objective measures have been trailed, few health care studies [13,14] have integrated log-based behavioral data with standardized questionnaires. To address this gap, our study proposes a dual-method evaluation framework that unites SUS scores with detailed behavioral engagement metrics, providing both perceived and observed dimensions of usability in caregiver-facing DT systems.

To address this gap, this study conducted a usability evaluation of an elderly digital twin (EDT) system designed to support informal caregivers in home care settings. In our previous work [18], we proposed a comprehensive EDT framework to assist caregivers in making informed decisions. To evaluate the feasibility and applicability of the framework, we developed a working EDT system prototype. Unlike prior studies that emphasized technical modeling or data integration, this study explicitly targets the informal caregiver perspective, in which usability is critical for adoption in daily home care. Our evaluation follows a dual-method approach with the following two objectives: (1) subjective evaluation of the usability of our proposed EDT system by assessing caregivers’ perceptions using the hugely popular SUS; (2) objective evaluation of usability by proposing a novel behavioral engagement metric that can be used in conjunction with the SUS.

We present a dual-method usability evaluation framework that combines log-based behavioral analytics (objective measurement) with a standardized SUS questionnaire (subjective measurement) [19]. This approach leverages the complementary strengths of objective behavioral data and subjective user feedback to provide a comprehensive view of the system’s usability. The log-based component captures detailed user behavior, including interaction patterns, feature usage, task completion pathways, error rates, and temporal usage characteristics. These metrics offer insights into real-world usage that may not be revealed through self-reported measures alone. In parallel, the SUS component provides standardized usability scores that enable benchmarking against established norms in health care applications and allow comparisons with other digital health interventions.

This dual-method approach directly addresses a noticeable gap in existing evaluation practices: the dominance of subjective methods with limited or superficial use of objective interaction data. Previous health care systems’ usability studies often relied on the SUS or combined it with a basic usage log [13,14,17], which failed to capture how systems are actually navigated and used in real practice. By integrating behavioral engagement metrics with subjective user perceptions, our study provides an evidence-based, user-centered, and replicable framework for evaluating digital health applications. Applied specifically to caregiver-facing interfaces for DTs of the older adults, this methodology not only fills the gap but also offers actionable insights to guide iterative design and ensure more effective, caregiver-friendly digital health systems. The remainder of this manuscript is organized as follows: Section 2 reviews related work, Section 3 outlines the methods, Section 4 presents the results, Section 5 offers a discussion, and Section 6 concludes the study.

### Related Work

The growing complexity of older adult care has driven the development of digital health technologies, including DT systems, to support clinical decision-making and informal caregivers. As these systems advance, ensuring their usability, particularly for nonprofessional caregivers, is critical for adoption and effectiveness. Previous studies have evaluated digital health technologies using subjective measures, such as the SUS or qualitative interviews. This section reviews usability studies and highlights the methodological gap addressed by our dual-method approach.

### Usability Evaluation Methods: Subjective Versus Objective

Usability evaluation methods fall into 2 primary categories: subjective and objective. Each category offers unique advantages and faces distinct limitations, making the choice between them or their combination critical for comprehensive usability assessment. SUS remains the most widely adopted usability instrument, valued for its brevity, reliability, and extensive benchmarking data [20,21]. Recent validation studies have reinforced SUS’s psychometric properties of the SUS. A validation study of voice user interfaces confirmed SUS’s usefulness across different interaction modalities. Additionally, researchers have developed item-level benchmarks for the SUS, allowing practitioners to interpret individual items when specific usability attributes require targeted assessment. These benchmarks enable a more granular evaluation while maintaining the scale’s standardized scoring advantage [20,22].

In addition to the SUS, several other standardized questionnaires serve specific evaluation contexts. The usability metric for user experience (UMUX) and its shortened variant, UMUX-LITE, offer alternatives that are more closely aligned with the ISO (International Organization for Standardization) 9241 definitions of usability. Recent psychometric evaluations suggest that the UMUX-LITER provides the closest correspondence to SUS scores when converted to comparable scales [23]. The NASA-TLX (National Aeronautics and Space Administration–Task Load Index) addresses perceived workload in complex, high-consequence environments, making it particularly valuable for health care, aerospace, and military applications. However, its complexity and administration time limit its usefulness for consumer product evaluations [24]. AttrakDiff occupies a unique position by measuring both the pragmatic and hedonic quality dimensions of the user experience. This dual focus allows for the evaluation of traditional usability metrics alongside emotional and aesthetic responses, although recent studies have raised concerns about translation reliability and cultural adaptation [25].

Subjective methods are easy to administer, resource-efficient, and standardized, enabling benchmark comparisons and effective communication among practitioners [26,27]. However, subjective measures have significant limitations. Social desirability bias is a primary concern, with users potentially providing responses they believe evaluators want to hear rather than their genuine perceptions. Cultural factors also influence responses, with different populations rather than actual performance potentially missing critical usability issues that users may not consciously recognize [25,26,28].

Objective evaluation centers on measurable performance indicators, such as task completion, time on task, and error analysis, which together provide insights into system effectiveness and efficiency [29-31]. Error analysis offers detailed insights into the usability problems. Error rates can be calculated globally (total errors divided by total attempts) and task-specific (errors per task opportunity), providing different perspectives on system performance. Modern approaches distinguish between error types and severity levels, enabling targeted improvements [29,31].

Clickstream analysis is a core component of behavioral log methods and has emerged as a powerful objective approach for tracking user navigation patterns, feature usage, and drop-off points. By capturing the sequence of interactions, it highlights problems that are invisible to traditional subjective metrics [32-34]. Advanced behavioral analytics extend this further by combining multiple interaction types, such as clicks, mouse movements, and page transitions, to generate comprehensive user journey maps. These methods are particularly valuable for detecting navigation bottlenecks and optimizing conversion paths in digital systems.

Objective methods generally provide concrete evidence for design decisions by revealing fine-grained interaction patterns that users may not consciously recognize or accurately report [35,36]. However, they also face implementation challenges: data collection and analysis can be complex and resource-intensive, and these methods may not directly capture user satisfaction or emotional responses, requiring supplementary subjective measures. Moreover, standardized interpretation frameworks for many objective metrics remain underdeveloped, complicating cross-study comparisons [26]. Recent health care usability studies have demonstrated effective hybrid implementation [37] and described a hybrid approach that satisfies both pragmatic development needs and academic research requirements. Their framework captures detailed behavioral data for intermediate iterations while enabling deeper qualitative analysis for academic dissemination. Contemporary usability evaluation is shifting toward integrated approaches that combine machine learning with behavioral subjective data analysis. Advanced statistical methods improve the correlation between objective performance and user satisfaction [38]. Recognizing that no single method offers a complete picture, the field increasingly adopts multimethod strategies that balance methodological strengths and limitations while maintaining practical feasibility [37].

### Clinical Relevance of Fitbit Sense 2 Data

Consumer wearables are increasingly used in digital health because they enable continuous, low-burden physiological monitoring outside clinical settings. Their clinical relevance, however, is parameter-specific rather than uniform. A systematic review of Fitbit devices found the strongest evidence for step counting under selected conditions, whereas energy expenditure, sleep, and some other measures showed less consistent accuracy and should be interpreted cautiously in health-related decision contexts [39]. A later meta-analysis of wrist-worn Fitbit sleep models reported that newer sleep-staging devices performed better than earlier motion-only models but still were not substitutes for polysomnography [40]. Because this study used Fitbit Sense 2, newer-generation evidence is also relevant: a prospective multicenter validation study of 11 consumer sleep trackers found that Fitbit Sense 2 showed moderate agreement with polysomnography for sleep-stage classification and competitive performance among wearables, including relatively strong performance in deep-stage detection [41].

Taken together, these findings support a measured role for Fitbit Sense 2 in caregiver-facing systems. Recent free-living validation of a newer Fitbit device against medical-grade references showed moderate to good agreement for daily steps, resting heart rate, respiratory rate, and some heart rate variability (HRV) measures but weaker agreement for oxygen saturation, indicating that some physiological channels are more dependable than others [39,41,42]. Accordingly, Fitbit Sense 2 data in the EDT are best viewed as supporting longitudinal trend monitoring, anomaly awareness, and caregiver-oriented decision support rather than diagnostic inference.

### Current Usability Evaluation Methods for Health Care Systems

As shown in Table 1, prior usability studies of digital health systems have relied on subjective methods, particularly the SUS, interviews, and qualitative feedback [13-15,17,43-48]. Only a few studies incorporated partial task-based or observational measures, and these were typically limited and not derived from detailed real-time interaction logs [13,14,45]. Moreover, studies focused on patients, older adults, or clinical professionals in institutional settings rather than informal caregivers in home-based care [16,17,44-48]. Synthesizing these studies reveals three major gaps: (1) heavy reliance on subjective measures without integrating detailed objective interaction logs, (2) limited focus on formal caregivers in home settings, and (3) superficial assessment of task behavior, where satisfaction or completion was reported but navigation patterns, retries, and feature usage were not captured. To address these gaps, this study integrates subjective usability perceptions (SUS) with objective system usage data (user activity logs), providing a more ecologically valid and holistic framework for evaluating caregiver-facing DT systems for older adults.

**Table 1.** Comparison of usability methods, target groups, and system features across related studies.

Target users	Method	SUS^a^	Log	Setting	Focus	Behavioral metrics	References
Informal caregivers	SUS, interview	No	No	Home	Stroke support	No	[43]
Older adults + Caregivers	SUS, interview	72.2	Partial	Home	Cognitive monitoring	Partial	[14]
Older adults + Caregivers	SUS, NASA-TLX	82.6	No	Home + Clinical	Heart Failure	Partial (observed)	[13]
Older adults	SUS, acceptability score	59.7	No	Clinical	Gait monitoring	No	[17]
Older adults	SUS, feedback	58.3	No	Lab + Clinical	Fall prevention	No	[15]
Patients	SUS	87.5	No	Clinical	Symptom tracking	No	[45]
Health care professional	SUS	69.2	No	Geriatric wards	Health monitoring	No	[16]
Older adults	SUS + Interview	78.8	No	Home	Home care platform	No	[46]
Patients + Clinicians	SUS + Feedback	86.8	No	Clinical	Trial data capture	No	[48]
Emergency staff	SUS + Interview	53.1	No	Clinical	Clinical information management	No	[47]
Our study (informal caregivers)	SUS + System usage logs	80.45	Activity logs	Home	Decision support in older adult care	Task metrics, user engagement score	—^b^

a SUS: System Usability Scale.

b Not applicable.

## Methods

### Study Design

This study evaluated the usability of the EDT system for supporting informal caregivers in home-based care. To provide a comprehensive assessment of user interaction, both subjective and objective measures were integrated. The following sections describe the study design, system features, participant recruitment, testing procedures, and analytical methods used to assess usability.

### Overall System Flow

**Figure 1.**
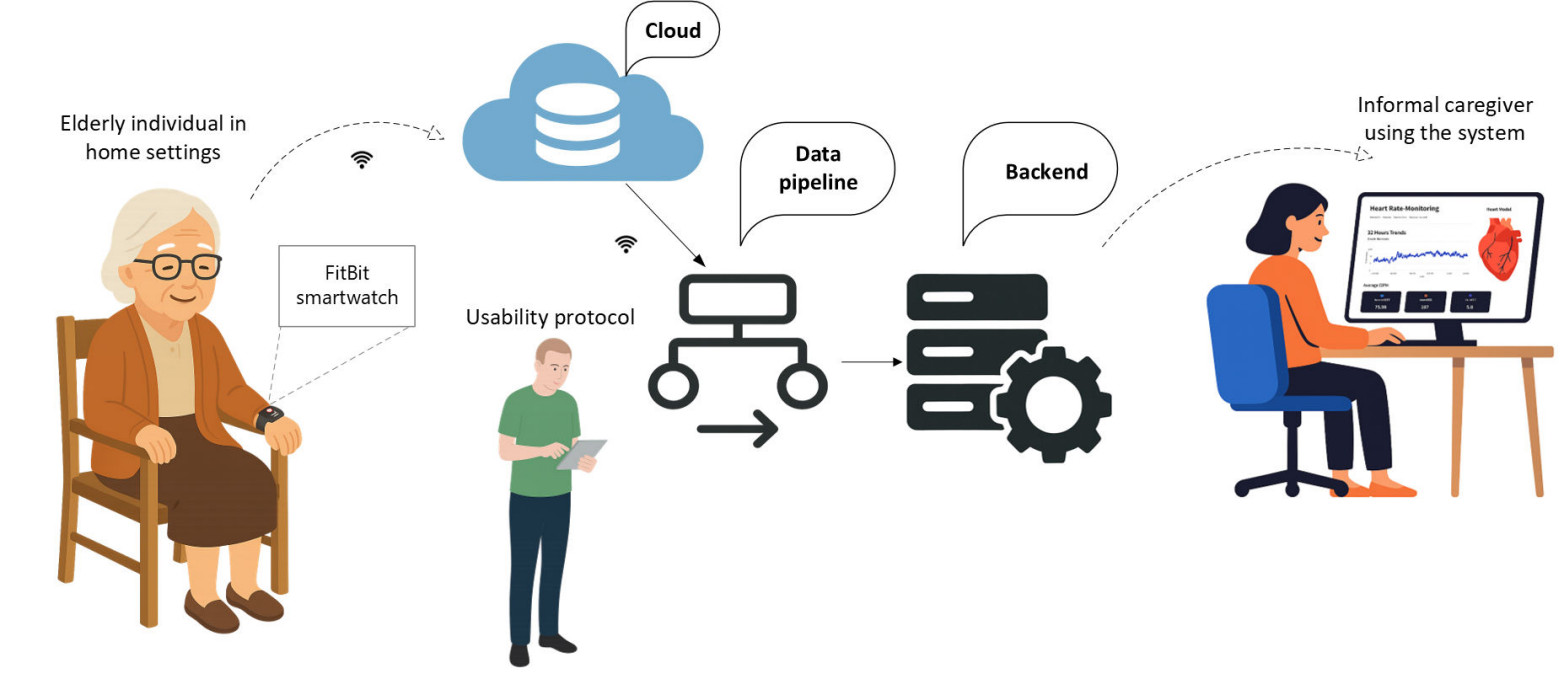
Illustration of the system usability session setup, showing data flow from a home-based older adult to a digital twin platform accessed by an informal caregiver.

**Figure 2.**
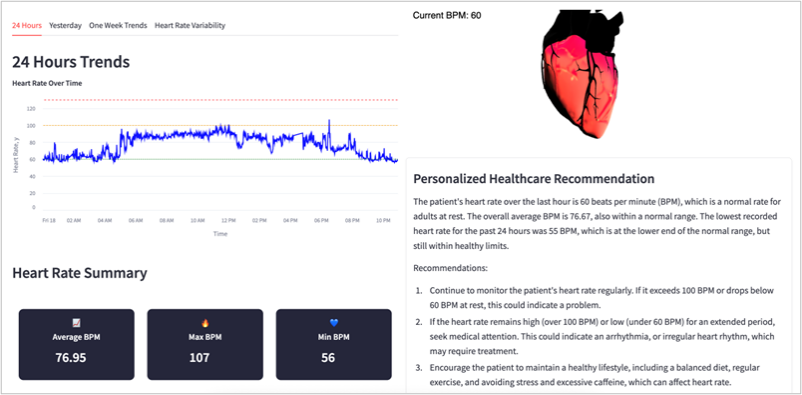
Cardiac Digital Twin model user interface.

**Figure 3.**
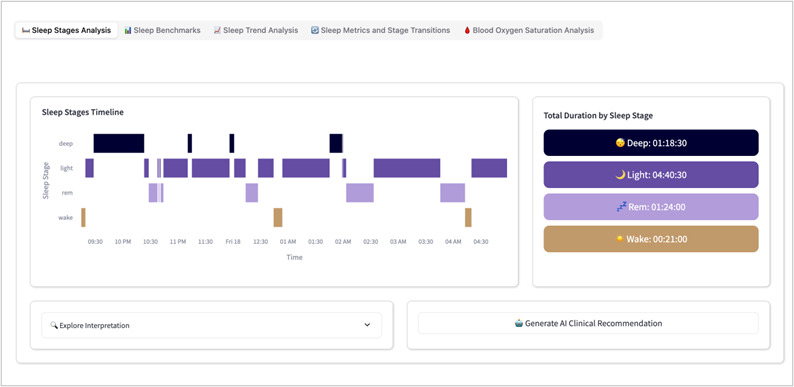
Sleep stages analysis user interface.

We used a cross-sectional observational design to evaluate the usability of the caregiver-facing EDT prototype. Figure 1 illustrates the system architecture and data flow during the usability sessions. Older adult participants wore a Fitbit smartwatch to collect physiological data, including heart rate, sleep, and activity measures. These data were transmitted to the cloud for secure transfer and preprocessing and then processed and stored by the back end for analysis and insight generation. Caregivers accessed the EDT system through computers, tablets, iPads, or smartphones, where they viewed real-time and historical health information, including digital biomarkers and artificial intelligence (AI)–generated recommendations. During the sessions, informal caregivers completed representative tasks such as exploring dashboards, reviewing AI-based recommendations, and navigating system features. User interactions were recorded through back-end logs, and caregiver feedback was collected using a questionnaire. Sample user interfaces of the system are presented in Figure 2 for cardiac DT and Figure 3 for sleep stage monitoring, while detailed system feature interfaces are provided in the Multimedia Appendix 1.

### System Features

**Figure 4.**
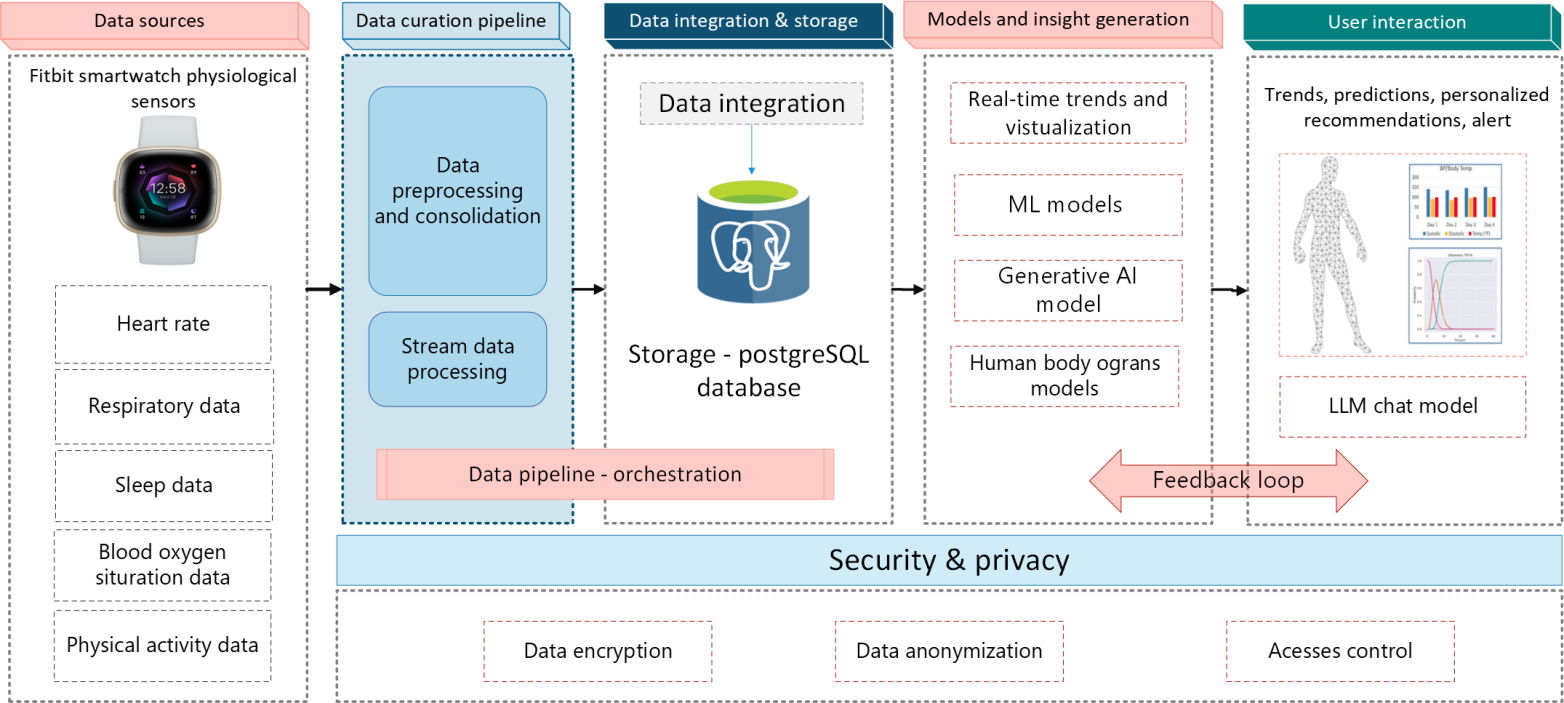
High-level architecture of elderly digital twin system. AI: artificial intelligence; LLM: large language model; ML: machine learning.

The EDT system evaluated in this study was designed to provide informal caregivers with real-time, personalized insights into the health status of older adults in their homes. The system follows a modular architecture with 5 core components: data sources, data curation pipeline, data integration and storage, models and insight generation, and user interaction, with a dedicated security and privacy layer and a dynamic feedback loop between the user and intelligent models, as shown in Figure 4.

### Data Sources

The system captures multimodal physiological data using a Fitbit smartwatch worn by older adults. These signals included heart rate, respiratory data, sleep log data, blood oxygen saturation (SpO_2_), and physical activity.

### Data Curation Pipeline

Incoming sensor data are streamed into an automated preprocessing module that handles data cleaning, synchronization, and consolidation across modalities. A dedicated stream processing engine prepares the data for integration and downstream analysis in real time. This stage ensures quality and consistency before the data are forwarded through an orchestrated pipeline.

### Data Integration and Storage

The processed data were stored centrally in a PostgreSQL database. This layer supports structured data storage and efficient querying for both real-time and retrospective analyses. Integration mechanisms ensure temporal alignment of physiological signals for model consumption.

### Models and Insights Generation

The EDT system comprises 2 main components: a Cardiac Digital Twin and a Sleep Monitoring Digital Twin, which monitor physiological signals and generate insights for caregivers. The EDT prototype was evaluated as a caregiver-oriented decision support research system rather than as a diagnostic tool, and the sensing device used in this study, Fitbit Sense 2, is not a Food and Drug Administration–approved device for clinical use. Cardiac Digital Twin tracks heart health in older adults through real-time heart rate monitoring, trend visualization, and HRV analysis. A 3D cardiac model simulates a beating heart with animation driven by current beats per minute. For continuous monitoring during data loss, a bidirectional long short-term memory (Bi-LSTM) model predicts beats per minute at 5-minute intervals. Bi-LSTM was selected because of its ability to capture temporal dependencies and improve physiological prediction accuracy. When activated, the system notifies the caregivers that synthetic predictions are being used. The Bi-LSTM model was trained in 579,486 heart rate records, achieving a mean squared error of 0.2944 and a mean absolute error of 0.3410. The reported model performance metrics are presented to characterize the technical behavior of the prototype components and should not be interpreted as evidence of clinical-grade validity.

The Sleep Monitoring Digital Twin processes multisensory physiological data to monitor sleep behavior. It analyzes patterns across sleep stages, including light, deep, rapid eye movement, and awake, and visualizes trends to support long-term observation. The system computes sleep quality metrics, such as efficiency, latency, and awakenings, using probabilistic models to map the stage transitions. These outputs enable the detection of sleep disruptions and behavioral anomalies linked to health decline in the older adult population. A long short-term memory model was developed to infer sleep stages from SpO₂ and heart rate data, chosen for its memory structure suitable for temporal dependencies in physiological time series. The model achieved 92% validation accuracy for 9125 sequences, maintaining sleep-tracking continuity. In this study, however, the sleep-monitoring model was included to enable the functional operation of the caregiver-facing EDT interface and was evaluated as part of the overall usability workflow rather than as a stand-alone clinically validated diagnostic model. A submodule monitored SpO₂ levels to detect hypoxemia, triggering alerts and large language model (LLM) feedback when oxygen saturation fell below the critical threshold. The LLM outputs are intended to support caregiver awareness and follow-up, rather than provide medical diagnosis or treatment advice.

To enhance real-time interpretability and caregiver empowerment, both DT models were supported by a fine-tuned GPT-4o LLM through instruction-based fine-tuning in caregiving. This LLM module delivers context-aware personalized feedback by interpreting physiological indicators, including abnormal heart rate patterns, HRV, disrupted sleep trends, sleep efficiency, latency, awakening, and hypoxemia through SpO_2_ monitoring. When the system detects anomalies such as elevated heart rate, low HRV indicating stress, oxygen saturation below thresholds, or deviations from sleep benchmarks, it triggers the LLM to generate clear, caregiver-facing recommendations. These responses are personalized based on recent health trends and are presented in an understandable language. LLM summarizes patterns and provides decision support recommendations for caregiver actions such as monitoring hydration or seeking clinical evaluation. This fine-tuned GPT-4o model translates complex sensor data into actionable guidance, strengthens decision support capabilities, and fosters informed caregiving responses.

In our previous study, the caregiver-facing recommendations were subjected to structured medical experts' evaluation across the dimensions of clinical appropriateness, usefulness, safety, and alignment with clinical guidelines, with overall positive ratings and substantial interrating agreement, supporting their use as decision support guidance in the present system [18]. To reduce the hallucinations, the GPT-4o model was developed using instruction-based fine-tuning in caregiving and grounded in structured physiological indicators from the EDT. The model was activated only when anomalies or threshold deviations were detected. The decision support recommendation generation was constrained through restricted prompting and limited output scope. Its outputs were designed as caregiver-friendly guidance and human-readable summaries of observed sensor patterns and not diagnosis or treatment advice. The system also used escalation language, such as advising clinical evaluation for concerning cases.

### User Interaction

The system provides a web-based interface for informal caregivers that is accessible through desktops, smartphones, and tablets. The dashboard enables navigation across modules such as heart rate trends, sleep analysis, SpO₂ monitoring, and alerts. This alert section presents LLM-generated alert messages and recommendations when abnormal physiological patterns or threshold violations are detected. Visualizations use color-coded indicators and summaries to reduce cognitive load. The GPT-4o chatbot further generates natural language feedback and recommendations based on health data and caregiver queries. Caregivers can review alerts, add notes, and interact with the system across devices, thus ensuring flexible and informed older adult care support.

### Feedback Loop for Adaptive Learning

The architecture incorporates a dynamic feedback loop based on caregiver responses and system logs. These feedback signals are used to retrain the detection thresholds, improve alert relevance, and fine-tune the language model for more accurate and useful recommendations.

### Security and Privacy

A dedicated privacy layer ensures compliance with data protection standards. It includes data encryption, access control mechanisms, and data anonymization to protect sensitive health data and support the deployment of ethical systems.

### Participants Recruitment

This study included 2 groups of participants: older adults and their informal caregivers. Six older adult participants were recruited to generate real-world physiological data for the validation of the monitoring function of the system. These individuals, aged 60‐85 years, were Thai nationals residing in home care environments and were cognitively and physically capable of using a Fitbit Sense 2 device. Patients with stable chronic health conditions were included in the study, while those with known skin sensitivities, cognitive impairments, or conditions interfering with the use of wearable devices were excluded.

Fifty informal caregivers were recruited for the usability evaluation using a purposive sampling strategy to ensure the representation of individuals actively engaged in home-based older adult care. Table 2 summarizes the demographic characteristics of the informal caregiver participants. Eligible caregivers were adults aged 18‐55 years with at least 3 months’ caregiving experience. To support meaningful interaction with the system, participants were required to demonstrate basic digital literacy (eg, prior use of smartphones or web apps) and the ability to navigate English-language interfaces. Screening interviews confirmed these criteria prior to enrollment. For better recruitment and participant comfort, all initial communications and consent discussions were conducted in the local Thai language with caregivers and older adult individuals. A local Thai assistant was engaged to explain the study procedure and provide step-by-step system guidance in Thai, ensuring clarity and inclusivity during the sessions. Caregivers received a stipend of 100 Thai Baht (average THB to US dollar exchange rate in July 2025: THB 1=US $0.03082; THB 100=US $3.082) to acknowledge their time and contribution. The Fitbit Sense 2 smartwatch used in this study was separately provided by the research team to all older adult participants.

**Table 2.** Demographic characteristics of informal caregiver participants.

Characteristic	Caregivers (n=50)
Age (years), mean (SD)	35.6 (7.6)
Age (years), range	18‐55
Age (years), group distribution, n (%)	
18‐25	3 (6)
26‐35	25 (50)
36‐45	15 (30)
46‐50	6 (12)
51‐55	1 (2)
Sex, n (%)	
Male	21 (42)
Female	29 (58)
Caregiving experience (years), n (%)	
<1	11 (22)
1‐3	21 (42)
4‐6	12 (24)
>6	6 (12)

### Ethical Considerations

The study protocol was reviewed and approved by the institutional review board of King Mongkut’s University of Technology Thonburi under reference number KMUTT-IRB-COA-2025‐048 on July 7, 2025. Written informed consent was obtained from all participants prior to data collection. Specifically, informed consent was secured from both the older adult participants and their informal caregivers before their involvement in the study. Caregiver participants received a small stipend of THB 100, equivalent to approximately US $3.08, as compensation for their time. This compensation was not linked to task performance, usability ratings, or study outcomes and was not considered coercive. All collected data were deidentified prior to analysis, and participant confidentiality was maintained throughout the study.

### Usability Evaluation Framework

**Figure 5.**
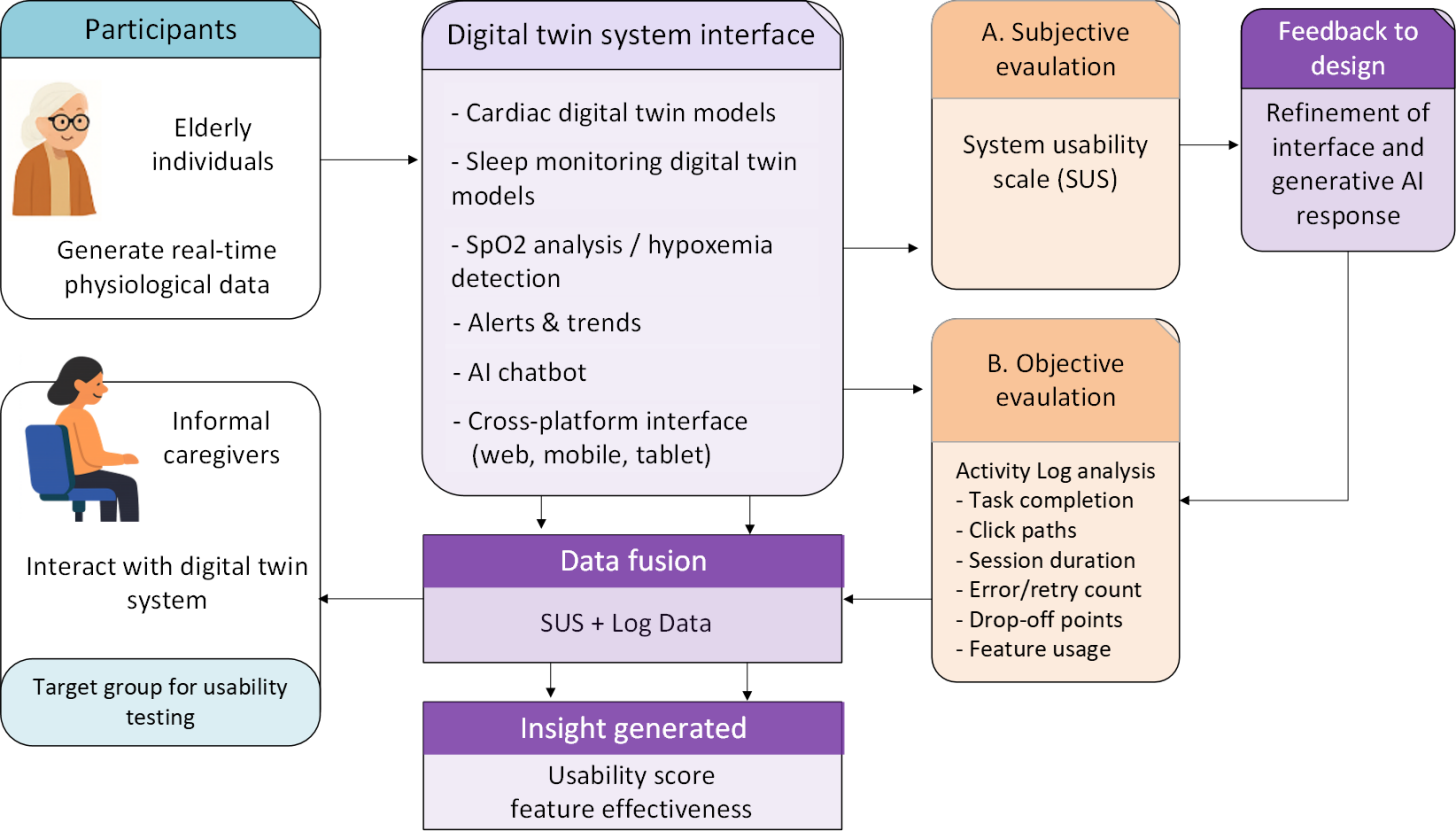
Usability evaluation framework for the elderly digital twin system. The framework integrates subjective (System Usability Scale) and objective (log-based) evaluation methods to assess system usability and inform design improvements. AI: artificial intelligence.

This study used a dual-method usability evaluation framework designed to capture subjective perceptions and objective behavioral interactions with the EDT system. The framework, illustrated in Figure 5, consists of 3 key components: participant roles, system interaction, and evaluation methods, integrated through a feedback-driven design loop. Two participant groups were involved: older adults who contributed real-time physiological data using a Fitbit Sense 2 smartwatch, and informal caregivers who interacted with the system to evaluate its usability. Usability was assessed using both subjective and objective methods. Subjectively, SUS was used to collect caregiver-reported feedback on ease of use, satisfaction, and overall system experience. Objectively, the activity log data captured granular interaction behaviors, including task completion rates, click paths, session duration, error rates, drop-off points, and module usage frequency. These 2 data streams were then fused to generate comprehensive usability insights. This fusion enabled us to assess both how users perceived the system and how they interacted with it.

### Data Collection

Data Collection was conducted over an 8-week period (July 7 to August 31, 2025) and involved 2 participant groups: older adults and informal caregivers. The older adult participants wore the Fitbit Sense 2 smartwatch in their home environments to generate real-time physiological data, including heart rate, respiratory rate, SpO_2_, sleep logs, and physical activity. Before commencing data collection, all older adult participants completed a brief health and comfort assessment to confirm their suitability for using a smartwatch. The survey gathered basic demographic information and screened for potential risks, such as allergies, history of skin irritation, relevant medical conditions, or wrist swelling, which could affect comfort or safety. Participants with contraindications were excluded. The results of the health and comfort screening are summarized in Table 3. This prescreening step ensured that the smartwatch could be used safely and comfortably during subsequent data collection.

**Table 3.** Health and comfort screening outcomes for older adult participants.

Screening item	Yes/No, n (%)
Allergy to smartwatch materials (silicone, metal, and adhesives)	0 (0)/6 (100)
History of skin irritation from wristband/watches/jewelry	0 (0)/6 (100)
Skin/medical conditions affecting smartwatch use	0 (0)/6 (100)
Swelling in wrist/hands	0 (0)/6 (100)
Physical activity	Daily: 3 (50), occasionally: 1 (16.7), and rarely: 2 (33.3)

The data were streamed to the system back end to validate the predictive analytics and monitoring capabilities of the EDT models under real-life conditions. Informal caregivers evaluated the system usability through guided interaction sessions using their smartphones, tablets, or computers. Each caregiver used the EDT system for 4 consecutive days, engaging with it for 20‐30 minutes daily. The SUS questionnaire was administered after the fourth day to capture the caregivers’ consolidated perception of usability after repeated interactions. This approach balanced ecological validity with participant burden: 4 days ensured sufficient system interaction while remaining feasible for busy caregivers. We also captured users’ behavioral data through system-generated activity logs, which were passively recorded through automatic instrumentation. The system obtained informed consent prior to log data collection, disclosing that no personally identifiable information was recorded. All logged data were anonymized and stored per institutional guidelines.

A total of 24 system tasks were predefined for the log data collection phase. These tasks were systematically constructed from the core functional modules of the EDT prototype, including heart monitoring, sleep tracking, SpO_2_ assessment, AI-driven health advice, and the AI chatbot assistant. The task set was designed to ensure structured coverage of the system’s principal functions during usability testing, so that all key user interactions could be consistently captured in the log analysis. Thus, the tasks were intended primarily to exercise the major features of the prototype rather than to directly reproduce naturally occurring caregiving workflows derived from interviews, observational studies, or clinical workflow analysis. Each task represented a meaningful user interaction within the prototype (eg, viewing 24-hour heart rate trends or checking sleep stage analysis), and each was assigned a unique code for consistent identification in the analysis, as detailed in Table 4.

**Table 4.** System tasks and associated codes used for feature usage analysis during the user activity log data collection.

Model and task	Logged behavior	End role	Associated objective metrics
Cardiac Digital Twin			
Request AI^a^ insights for real-time heart rate	Click on AI insights	AI output shown	TC^b^, ToT^c^, FU^d^
Observe real-time heart rate dashboard	Open live HR^e^ screen	Exit/switch page	TC, ToT, RR^f^, NF^g^, FU
View 24-hour heart rate history	Open 24-hour chart	Exit/switch page	TC, ToT, NF, TAR^h^, FU
View 7-day heart rate trends	Open 7-day HR trends	Exit/switch page	TC, ToT, FU
View 24-hour heart rate summary	Open HR summary panel	Exit/switch page	TC, ToT, TAR, FU
Observe heart rate variability dashboard	Open HRV^i^ panel	Exit/switch page	TC, RR, ToT, FU
Request AI insights on heart rate variability	Click AI for HRV	AI output shown	TC, FU, CTR^j^
Sleep Monitoring Digital Twin			
View last night’s sleep quality	Open sleep summary	Exit/switch page	TC, NF, TAR, ToT
Request AI advice for abnormal deep sleep	Click AI for deep sleep	AI output shown	TC, FU, CTR, ToT
Request AI advice for low light sleep	Click AI for light sleep	AI output shown	TC, FU, CTR, ToT
Request AI advice for REM^k^ sleep levels	Click AI for REM sleep	AI output shown	TC, FU, CTR, ToT
View sleep metrics dashboard	Open sleep metrics	Exit/switch page	TC, ToT, TAR
View sleep stage timeline	Open stage timeline	Exit/switch page	TC, NF, CTR, FU
View sleep benchmark reference	Open benchmark panel	Exit/switch page	TC, NF, TAR
Review sleep benchmark interpretation	Open benchmark explanation	Exit/switch page	TC, ToT, RR, FU
Request AI advice on sleep benchmark	Click AI for benchmark	AI output shown	TC, session duration, TAR, FU, ToT, CTR
Open and generate sleep trend report	Open/generate report	Report shown	TC, session duration, TAR, FU
View weekly sleep log	Open weekly sleep log	Exit/switch page	TC, NF, TAR, FU
View sleep fragmentation and stage transitions	Open fragmentation view	Exit/switch page	TC, NF, ToT, FU
Request AI advice on sleep fragmentation	Click AI for fragmentation	AI output shown	TC, FU, session duration, ToT
SpO_2_ Monitoring			
View SpO_2_ analysis dashboard	Open SpO_2_ analysis	Exit/switch page	TC, ToT, NF, FU
Observe real-time SpO_2_ for hypoxemia monitoring	Open live SpO_2_ screen	Exit/switch page	TC, RR, TAR, FU
Request AI advice on SpO_2_ abnormalities	Click AI for SpO_2_	AI output shown	TC, FU, CTR, ToT
AI Assistant			
Submit query to AI health chatbot	Enter/send prompt	Response shown	TC, session duration, RR, CTR, TAR, FU

a AI: artificial intelligence.

b TC: task completion.

c ToT: time on task.

d FU: feature usage.

e HR: heart rate.

f RR: retry rate.

g NF: navigation frequency.

h TAR: task abandonment rate.

i HRV: heart rate variability.

j CTR: click-to-task ratio.

k REM: rapid eye movement

The mapping tasks for objective metrics were derived from the functional characteristics of each task. Metrics such as task completion and time on task were applied universally as core usability indicators. The retry rate was assigned to tasks with likely repeated attempts in monitoring and chatbot interactions. Feature usage was linked to AI-driven features, demonstrating user engagement beyond basic monitoring. Navigation frequency was applied to tasks involving timelines and log exploration. Session duration was associated with tasks requiring extended engagement, such as report generation. The click-to-task ratio was included for tasks with multiple interaction steps. The task abandonment rate was assigned where drop-offs were likely, such as when viewing summaries and reports. This mapping ensures the replicability of linking system interactions to the 8 objective usability metrics.

To ensure that the objective usability measures reflected actual user behavior, each logged task was operationalized as a concrete interface-level action. Tasks such as view and observe were defined as opening and accessing a specific dashboard, chart, or panel until the user exited or switched pages. AI-related tasks were defined as explicit user-triggered actions, such as clicking an AI insight or advice function or submitting a chatbot query, with completion recorded when the system response was displayed. In Table 4, open indicates that the user accessed a dashboard, chart, panel, or report. Click AI indicates an explicit user request for AI-generated insights or advice. Exit/switch page indicates that the user left the target screen or moved to another module. AI output shown indicates successful system generation and display of the requested response.

### Data Analysis

#### User Activity Log Analysis

**Figure 6.**
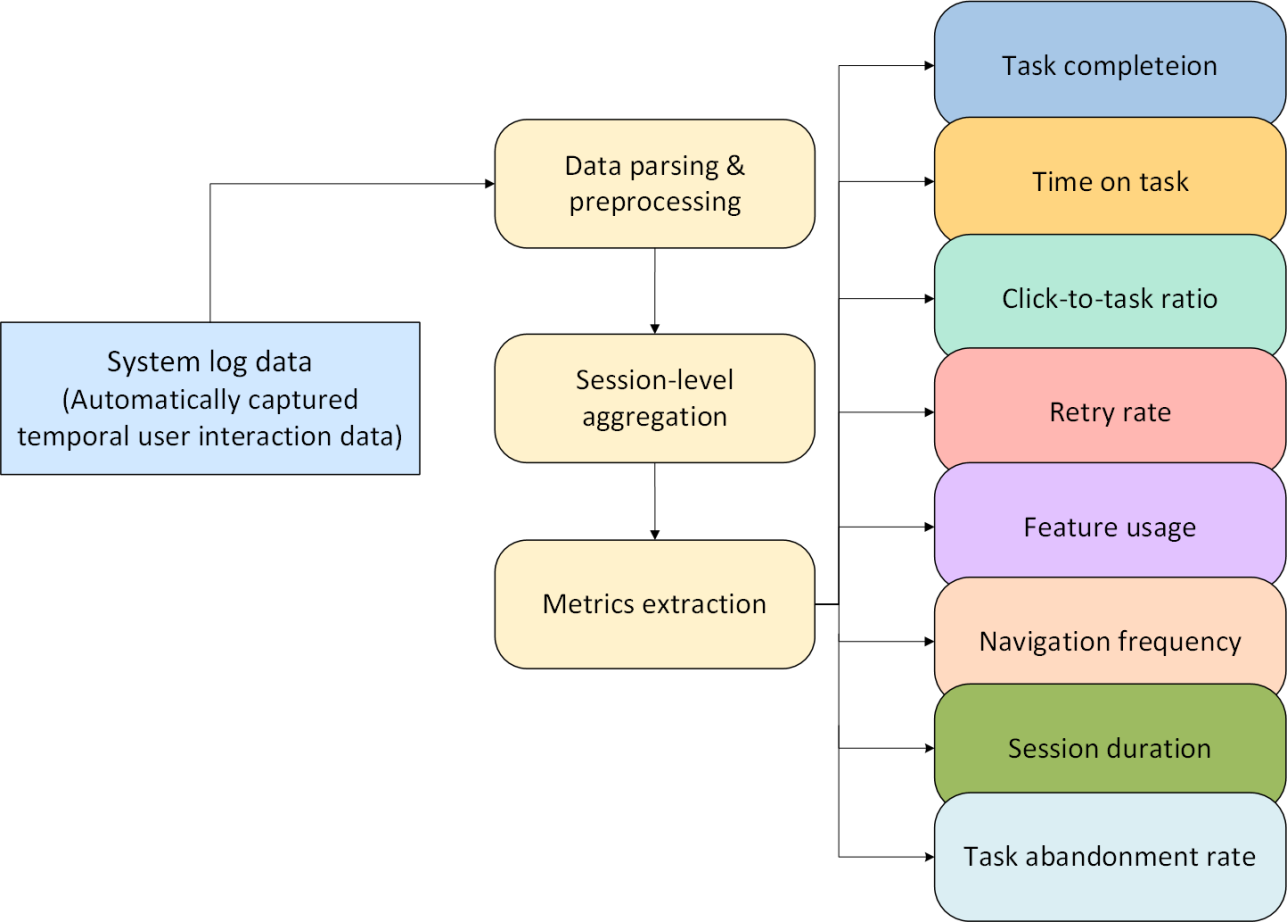
Sequential steps for transforming raw log data into quantified usability measures.

User activity logs were extracted from the system back end for all caregiver interaction sessions that were conducted during the study period. Each log entry included a time stamp, session identifier, user identifier, task name, action type (eg, started, completed, error, retry, and abandoned), task duration (in seconds), navigation events, and click events. As shown in Figure 6, these raw entries were processed at the session-level metrics, aggregated across sessions for each user, and normalized by relevant totals (eg, total tasks started) to enable cross-user comparisons.

#### Metrics Computation

The raw interaction logs were parsed and preprocessed, aggregated at the session level, and used for metric extraction. From this pipeline, key objective behavioral indicators were computed, including task completion (TC), time on task (ToT), click-to-task ratio (CTR), retry rate (RR), feature usage (FU), navigation frequency (NF), session duration, and task abandonment rate (TAR). TC is the percentage of started tasks that were completed. This metric was calculated using equation 1.


(1) $$TC\left(\%\right)=\frac{C_{i}}{S_{i}}\times 100$$

where *C*_*i*_ is the number of tasks of user *i* with completed status and *S*_*i*_ indicates the number of tasks with started status of user *i*. ToT measures how long it takes a user to begin or complete a task once they start interacting with the system. It is defined as:


(2) $$ToT=\sum_{i=1}^{n}\frac{d_{i}}{n}$$

where *d*_*i*_ is the duration of the *i* − *th* completed task and *n* is the number of completed tasks. The RR measures how often users had to repeat a task before succeeding. For each task, if a user has multiple STARTED events before success, everything after the first event is a retry, defined as follows:


(3) $$RR_{i}=\frac{r_{i}}{a_{i}}$$

where *r*_*i*_ is the number of retries for user *i* repeated a task after a failed attempt (errors, abandoned, and reclicks) and *a*_*i*_ the total number of tasks the user initiated. FU counts the interaction of users per system feature and was calculated as, FU_*x*_ = *N*_*x*_, where, *N*_*x*_ is the count of feature *x* in the navigation logs. Session duration was calculated as follows:


(4) $$SD=t_{end}-t_{start}$$

where *t*_start_ and *t*_end_ are the first and last actions in a session, respectively. The TAR is the percentage of tasks started but never completed and is defined as follows:


(5) $$\text{TAR}(\%)=\left(\frac{A}{S}\right)\times 100$$

where *A* is abandoned tasks. CTR is the number of clicks per completed task, defined as CTR = *K*/*C*, where *K* is the click event. NF is the number of times each navigation step/module is used, defined as NF_*x*_ = *N*_*x*_. All metrics were calculated per session and aggregated for user and cross-user analysis. Table 5 presents the descriptive statistics of the 8 objective usability metrics collected from caregivers’ interactions with the EDT system.

**Table 5.** Descriptive statistics of objective usability metrics.

Metric	Minimum	Maximum	Mean (SD)	
TC^a^ (%)	82.93	98.02	94.08 (4.10)	
RR^b^	0.00	1.00	0.25 (0.28)	
ToT^c^ (seconds)	39.20	175.67	89.16 (21.97)	
TAR^d^ (%)	0.00	14.13	2.66 (2.87)	
CTR^e^	0.12	1.32	0.48 (0.22)	
NF^f^	5.00	12.00	11.02 (1.39)	
Session duration (minutes)	36.97	171.90	101.59 (30.17)	
FU^g^ (event count)	52	98	54 (13.4)	

a TC: task completion.

b RR: retry rate.

c ToT: time on task.

d TAR: task abandonment rate.

e CTR: click-to-task ratio.

f NF: navigation frequency.

g FU: feature usage.

To establish the relationship between objective usability metrics derived from user activity logs and the dimensions of SUS, we mapped each metric to the SUS factors originally proposed by [49,50]. Their analysis suggested that the SUS can be interpreted as comprising 2 correlated but distinct dimensions: usable (covering items related to effectiveness, efficiency, and overall ease of use) and learnable (covering items reflecting the ease of learning and initial onboarding, specifically items 4 and 10).

The mapping of objective usability metrics to the SUS dimensions is presented in Table 6. In this study, we adopted the 2-factor structure of SUS (usability and learnability) as proposed by Borsci et al [49] and Lewis and Sauro [50], which is the most widely validated and parsimonious model compared with alternative factor structures. In this mapping, performance-related measures such as task completion, efficiency, engagement, and error-related indicators are aligned with the usability factor, as they reflect effectiveness and overall ease of use. In contrast, retry-related measures are aligned with the learnability factor, as they directly capture the effort required for users to acquire proficiency during initial interactions. This mapping provides a conceptual bridge between objective behavioral-based metrics and subjective usability perceptions, ensuring that activity log analysis contributes to the broader usability assessment framework represented by Borsci et al [49] and Lewis and Sauro [50].

**Table 6.** The definitions of the usability metrics derived from system activity logs.

Objective metric	Relevant usability aspect (from literature)	Mapped SUS factor (Lewis and Sauro [50])	References
TC^a^	Effectiveness (core usability outcome)	Usable (items 1, 3, 5, 7, 8, and 9)	[49-52]
ToT^b^	Efficiency	Usable (efficiency items contribute to overall ease-of use perceptions)	[49,50,53]
RR^c^	Learnability (ease of initial learning, errors before success)	Learnable (items 4 and 10: technical support and things to learn)	[49,50,52]
FU^d^	Discoverability (linked to usability breadth)	Usable (items 5 and 9: confidence and integration)	[49,50,54]
Session duration	Engagement	Usable (general usability experience, confidence, ease, and satisfaction)	[49,50,52]
TAR^e^	Usability issues/unclear flow	Usable (same rationale as drop-off points)	[49,50,55]
CTR^f^	Efficiency	Usable (efficiency dimension of usability)	[49,50,52]
NF^g^	User flow analysis	Usable (task integration, item 5 “functions well integrated”)	[49,50,56]

a TC: task completion.

b ToT: time on task.

c RR: retry rate.

d FU: feature usage.

e TAR: task abandonment rate.

f CTR: click-to-task ratio.

g NF: navigation frequency.

### User Engagement Based on Composite Behavioral Metrics

To capture a more holistic measure of user interaction with the EDT system, we computed a user engagement score (UES) by incorporating multiple behavioral indicators that were derived from the system logs. Specifically, 6 objective metrics were included: feature usage, average session duration, session frequency, task completion rate, error rate, and navigation diversity. Each captures a distinct aspect of usability: task efficiency, effectiveness, temporal aspects, and behavioral diversity [51-54,56]. This composite scoring approach is essential for usability studies because it provides a multidimensional perspective on user interaction. These holistic measures are recognized as valid indicators of sustained adoption and user satisfaction [57,58]. To derive the composite engagement score, we initially extracted 8 metrics from the system log. Because not all metrics uniquely represented user engagement, we retained 6 core metrics that directly reflected the breadth, depth, and effectiveness of user interactions. These metrics included FU, ToT (seconds), total session duration (seconds), TC, error count, and NF. Each metric was then normalized to a (0, 1) scale using the maximum value observed in the dataset. The normalized values were subsequently combined using equal weights to produce a composite engagement score, as presented in equation 5.


(6) $${Eng}_{i}=\frac{\alpha F_{i}+\beta S_{i}+\gamma R_{i}+\delta C_{i}+\epsilon E_{i}+\zeta D_{i}}{\alpha +\beta +\gamma +\delta +\epsilon +\zeta }$$

where *F*_*i*_ is the normalized feature usage for user *i*, *S*_*i*_ indicates normalized session duration, *R*_*i*_ is the normalized ToT, *C*_*i*_ shows the normalized values of TC, *E*_*i*_ is normalized error rate, *D*_*i*_ is the normalized navigation diversity, and α, *β*, γ, δ, ε, and ζ symbols show the weights. All weights were set to be equal (α = *β* = γ = δ = ε = ζ = 1) so that each engagement dimension contributed uniformly to the composite score. An equal-weight additive formulation was selected as a pragmatic postnormalization approach to combine the engagement metrics while avoiding bias or subjective prioritization in the absence of empirical evidence regarding their relative importance. Prior literature on composite scoring has similarly used unit weights as a baseline when neither theoretical nor empirical justification exists for different weighting [59-61]. Because formal testing of interdependence or dimensional structure among the component metrics was not performed in this study, the resulting engagement score should be interpreted as an operational composite summary rather than a formally validated latent construct. For interpretability, k-means clustering with *k*=3 was then applied to classify users into low-, medium-, and high-engagement groups.

### The SUS

The usability of the EDT system was evaluated using the SUS, a standardized 10-item questionnaire widely used to assess the perceived usability of interactive systems. Each item was rated on a 5-point Likert scale ranging from 1 (strongly disagree) to 5 (strongly agree). SUS data were collected from 50 informal caregivers. To compute individual SUS scores, responses to odd-numbered items were scored as the participant’s rating minus 1, whereas responses to even-numbered items were scored as 5 minus the rating. The adjusted item scores were summed and multiplied by 2.5, yielding total SUS scores ranging from 0 to 100. In addition, item-level descriptive statistics were computed for all 10 items. Internal consistency was evaluated using Cronbach α [62] after reverse-scoring the negatively worded items so that higher scores consistently indicated greater perceived usability, as defined in equation 6.


(7) $$\alpha =\frac{k}{k-1}\left(1-\frac{\sum_{i=1}^{k}\sigma_{i}^{2}}{\sigma_{T}^{2}}\right)$$

where *k* is the number of items, $\sigma_{i}^{2}$ is the variance of each individual item, and $\sigma_{T}^{2}$is the variance of total score (sum across all items).

### Correlation and Regression Analysis of Engagement and Perceived Usability

To investigate the relationship between the composite behavioral metric and perceived usability, we first examined the correlation between the UES and the SUS. The normality of both variables (UES and SUS) was tested using the Shapiro-Wilk test. As SUS scores deviated from normality, Spearman rank correlation (ρ) was selected as the primary measure of association, and Pearson correlation (*r*) was also reported for completeness. Bootstrapped 95% CIs were computed to quantify the precision of the correlation estimates. Following the correlation analysis, we conducted a simple linear regression to test whether the UES predicts the SUS. The regression was specified as follows:


(8) $${SUS}_{i}=\beta_{0}+\beta_{1}\cdot {UES}_{i}+\epsilon_{i}$$

where SUS_*i*_ is the usability score for user *i*, UES_*i*_ is the engagement score, *β*_0_ is the intercept, *β*_1_ is the regression coefficient, and ε*_i_* is the error term. Model fit was evaluated with *R*^2^, F-statistics, and coefficient significance. This 2-step approach enabled us to first establish the strength and direction of the association between user engagement and usability and then the predictive power of the UES for the SUS, thereby linking objective behavioral data with subjective ratings.

## Results

### Overview

This study assessed the EDT system usability among informal caregivers by analyzing objective user interactions and subjective perceptions of usability (SUS). Three aspects were analyzed: (1) SUS survey results reflecting perceived usability, (2) patterns of composite UESs from system logs, and (3) the relationship between user engagement and usability using correlation and regression analyses. These findings address the study aim of assessing EDT system usability using objective behavioral metrics and subjective measurements and examining whether behavioral engagement indicates perceived system usability.

### SUS Results

**Figure 7.**
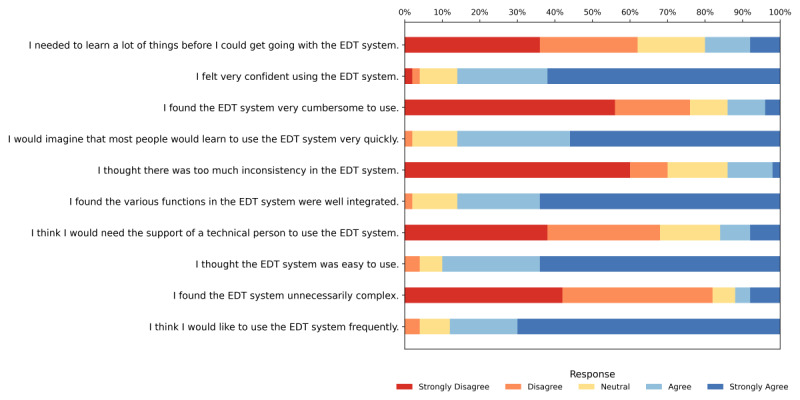
Distribution of responses to the 10 System Usability Scale items among caregivers (n=50). Percentages are shown on the top axis (0%‐100%). EDT: elderly digital twin.

Item-level response patterns for the 10 SUS items are presented in Figure 7, with descriptive statistics shown in Table 7. Overall, caregivers responded positively to the positively worded items and showed low agreement with the negatively worded items, indicating favorable usability perceptions of the EDT system. Higher mean scores were observed for confidence in using the system, integration of system functions, and intention to use it frequently, whereas lower engagement on negatively phrased items suggested limited perceived complexity, inconsistency, and need for technical support. The SUS also demonstrated strong internal consistency, with a Cronbach α of 0.87, indicating reliable measurement of perceived usability.

**Table 7.** Item-level descriptive statistics for the 10 System Usability Scale questions.

Item	Mean (SD)	Minimum	Maximum
Q1	4.54 (0.8)	2.0	5.0
Q2	1.96 (1.18)	1.0	5.0
Q3	4.50 (0.79)	2.0	5.0
Q4	2.18 (1.26)	1.0	5.0
Q5	4.48 (0.79)	2.0	5.0
Q6	1.86 (1.20)	1.0	5.0
Q7	4.40 (0.78)	2.0	5.0
Q8	1.86 (1.20)	1.0	5.0
Q9	4.42 (0.91)	1.0	5.0
Q10	2.30 (1.30)	1.0	5.0

The overall SUS scores (N=50) ranged from 42.5 to 100, with a mean of 80.45 (SD 17.6), indicating a generally favorable perception of usability by caregivers. As shown in Table 8, the SUS scores varied descriptively across the caregiving experience levels. Caregivers with more than 6 years of experience reported the highest mean score (mean 86.7, SD 21.8), followed by those with 1‐3 (mean 83.5, SD 14.5) years and 4‐6 (mean 81.0, SD 16.8) years of experience. Caregivers with less than 1 year of experience reported the lowest mean score (mean 70.9). A 1-way ANOVA was used to test whether these differences were statistically significant. Although the descriptive pattern suggested higher usability perception among more experienced caregivers, the ANOVA results were not statistically significant (*F*_3,46_=1.58; *P*=.208, η²=0.093). The effect size indicates that approximately 9.3% of the variance in the SUS scores was explained by caregiving experience, reflecting a small to moderate effect.

**Table 8.** Mean System Usability Scale scores by caregiving experience and age group.

Category (group)	Mean (SD) SUS^a^ score^b^
Caregiving experience (years)	
<1	70.9 (20.3)
1‐3	83.5 (14.5)
4‐6	81.0 (16.8)
>6	86.7 (21.8)
Age group (years)	
18‐25	80.8 (17.0)
26‐35	80.8 (16.1)
36‐45	77.0 (21.6)
46‐50	83.3 (18.4)
51‐55	92.5 (0.0)

a SUS: System Usability Scale.

b Overall mean score 80.45 (SD 17.65).

SUS scores also showed descriptive variation across age groups, as presented in Table 8. Caregivers aged 51‐55 years reported the highest mean score (mean 92.5, SD 0.0), while those aged 36‐45 years reported the lowest (mean 77.0, SD 21.6). Younger caregivers (aged 18‐25 and 26‐35 years) reported similar mean scores (mean 80.8), and caregivers aged 46‐50 years also rated usability favorably (mean 83.3, SD 18.4). However, 1-way ANOVA found no statistically significant differences across age groups (*F*_4,45_=0.399; *P*=.808, η²=0.034). This small effect size indicates that age accounted for only approximately 3.4% of the variance in the SUS scores. Thus, age did not meaningfully influence caregivers’ evaluations of the system. Overall, the mean SUS score of 80.45 exceeded the commonly cited 68-point benchmark and fell within the “excellent” range (≥80), showing that caregivers evaluated the EDT system very favorably.

**Figure 8.**
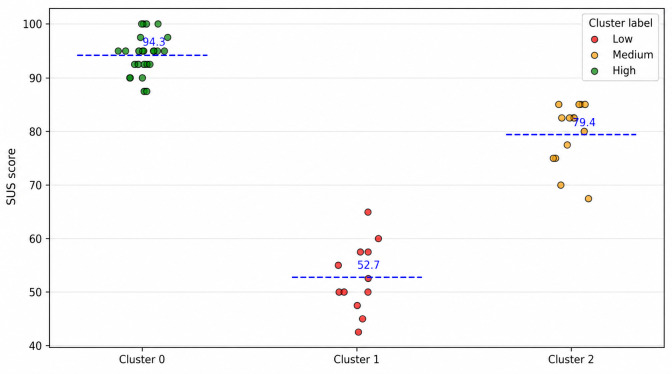
K-means clustering of SUS (N=50) into low, medium, and high groups. Dashed lines indicate cluster centroids. SUS: System Usability Scale.

Beyond demographic differences, we performed k-means clustering to classify caregivers’ SUS scores into 3 groups: low (mean 52.7), medium (mean 79.4), and high (mean 94.30). As shown in Figure 8, the clusters clearly distinguished participants with below-average usability perceptions from those who reported excellent usability. Most caregivers fell into the medium to high clusters, reinforcing the overall favorable evaluation of the system, while a smaller subgroup reflected lower usability perceptions, indicating areas for targeted improvements.

### User Engagement Based on Composite Behavioral Metrics Results

**Figure 9.**
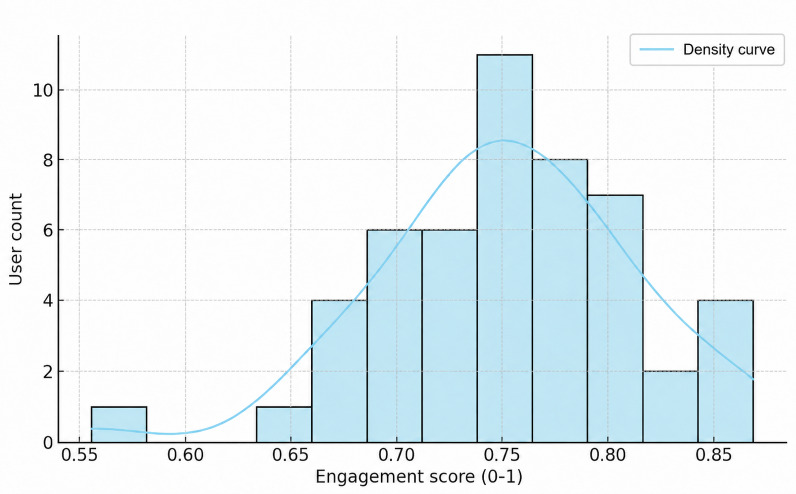
Distribution of composite engagement scores across users.

The composite UES was computed for all participants based on normalized behavioral metrics. The scores ranged from 0.55 to 0.86, with the majority of users clustering between 0.70 and 0.80, as shown in Figure 9. The distribution was slightly skewed toward higher engagement, indicating that most participants interacted consistently with the EDT system features, whereas only a few exhibited lower engagement levels.

**Figure 10.**
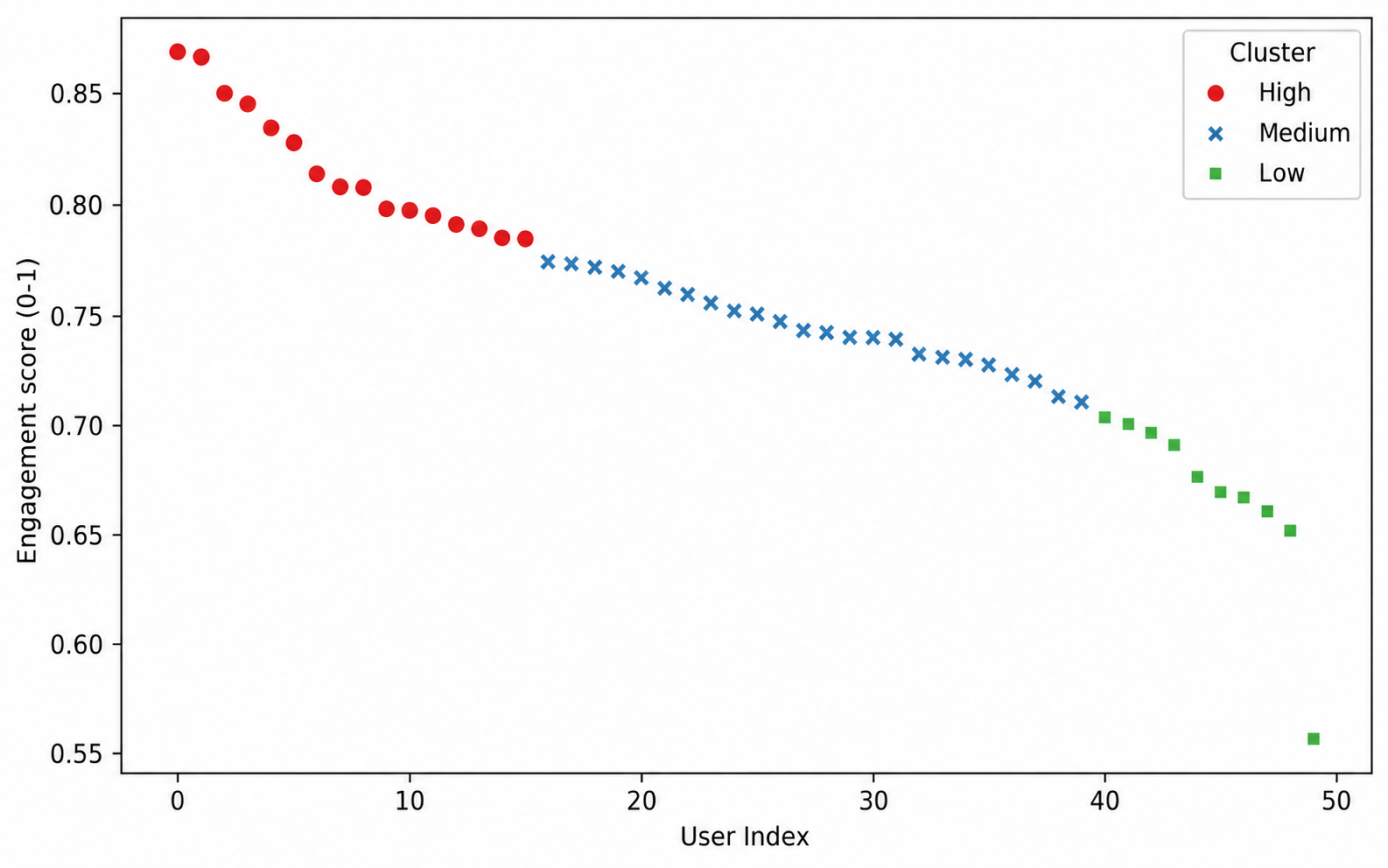
Composite user engagement tiers using k-means clustering (*k*=3).

For interpretability, users were classified into 3 engagement tiers (low, medium, and high) using k-means clustering (*k*=3). The results showed that most users belonged to the medium engagement group, with smaller proportions in the high- and low-engagement tiers, as shown in Figure 10. This distribution indicates distinct user subgroups, with strong engagement among highly engaged users and lower, yet observable, interaction among the least engaged. Overall, the pattern suggests that the EDT system supported sustained interaction for most users.

### Correlation and Regression Results for Engagement and Perceived Usability

**Figure 11.**
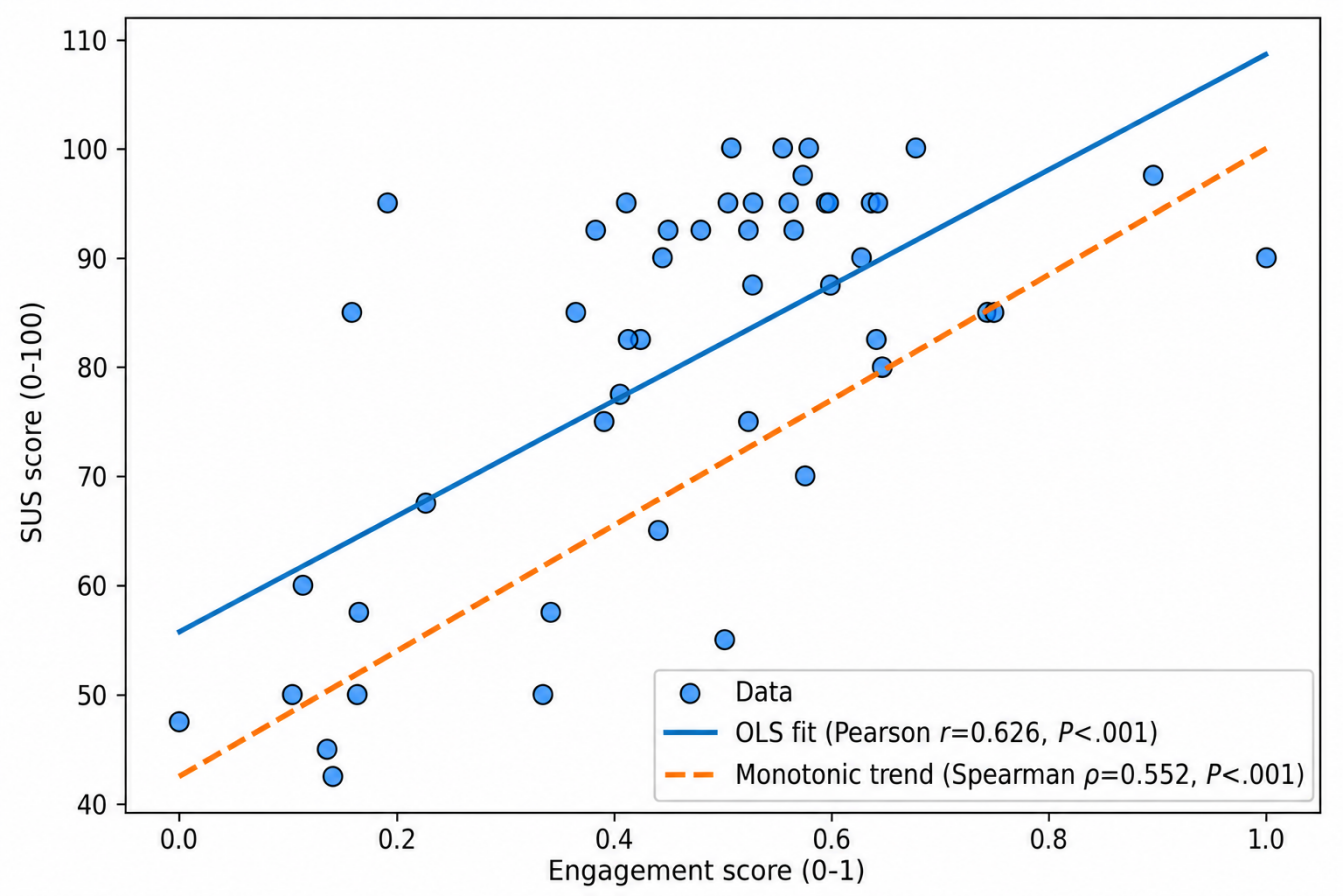
Scatterplot of engagement and SUS scores with Pearson linear fit (solid line) and Spearman monotonic trend (dashed line). OLS; ordinary least squares SUS: System Usability Scale.

Building on the relationship between the UES and SUS, we examined how user behavioral measures can predict the SUS of the EDT system. As shown in Table 9, the regression analysis further demonstrated that UES significantly predicted usability scores (*F*_1, 48_=31.00, *P*<.001), explaining 39.2% of the variance (*R*^2^=0.392). Engagement was a positive predictor (*β*=52.94, *t*_48_=5.57; *P*<.001), indicating that higher engagement was associated with higher SUS ratings. Figure 11 illustrates the correlation between SUS and UES.

**Table 9.** Correlation between System Usability Scale and composite user engagement scores.

Analysis and variable	Statistics (r/p)/*β* (coefficient)^a^	*P* value	95% CI	*R* ^2^	SE	*t* value
Correlation						
Pearson *r*	0.626	<.001	0.417-0.778	—^b^	—	—
Spearman ρ	0.552	<.001	0.311-0.734	—	—	—
Regression						
Intercept	55.73	<.001	45.97-65.49	—	4.86	11.48
UES (*β*)	52.94	<.001	33.82-72.05	0.392	9.51	5.57

a *r* indicates the Pearson correlation coefficient; ρ indicates the Spearman rank correlation coefficient; *β* indicates the regression coefficient.

b Not available.

## Discussion

### Principal Findings

This study found that the EDT system achieved generally positive usability outcomes among informal caregivers based on both subjective perceptions and objective behavioral metrics. SUS results suggested favorable perceived usability, and user activity logs reflected meaningful engagement with system features. The observed association between SUS and UES further indicates that behavioral metrics may complement self-reported usability measures in evaluating caregiver-facing digital health systems.

### Usability Evaluation Outcomes

The EDT system attained a mean SUS score of 80.45, exceeding the widely accepted threshold of 68 for above-average usability [19]. A score above 80 is typically interpreted as excellent usability [63]. In this sample, these results suggest that the participating informal caregivers perceived the EDT interface as relatively easy to learn and efficient to use. The composite UES derived from detailed activity logs was positively correlated with the SUS (Pearson *r*=0.626, Spearman ρ=0.552). The significant correlation between UES and SUS highlights the complementary nature of behavioral and perceptual usability measures. While the SUS captures caregivers’ subjective impressions of the system’s ease of use and satisfaction, the UES quantifies the depth and quality of their actual interactions. The strength of the observed correlation between the UES and the SUS can be interpreted as moderate to strong, where the correlation between performance and satisfaction metrics generally ranges from 0.38 to 0.70 and often does not exceed 0.7 [23,64].

Beyond this association, regression analysis demonstrated that UES was a meaningful predictor of the SUS, explaining 39.2% of its variance. In the regression model, the *F*-test result (*F*_1, 48_=31.00, *P*<.001) indicated that the model with UES as a predictor of SUS was statistically significant overall. The intercept (constant = 55.73) represents the predicted SUS value when UES equals zero; because zero engagement was not observed in the study data, this value should be interpreted as a model constant rather than a substantively meaningful estimate. Engagement score (*β*=52.94, *P*<.001) indicates that for every 1-point increase in UES (on its 0‐1 scale), SUS is predicted to increase by about 53 points. The coefficient of determination (*R*²=0.392) indicated that 39.2% of the variance in SUS was explained by UES. Because the model included only 1 predictor, this value is also consistent with the squared Pearson correlation between UES and SUS. The adjusted *R*^2^ value of approximately 0.38 suggests a comparable level of explained variance after accounting for sample size. Although much of the remaining variance is likely attributable to other factors influencing perceived usability, these results support a meaningful association between behavioral engagement and perceived usability within this sample.

In human-computer interaction research, behavioral predictors typically explain only a partial variance in subjective satisfaction metrics. Studies have shown that behavioral log data account for 30%‐40% of the variance in perceived usability, with the remainder due to unobserved factors [65,66]. This aligns with strong task performance metrics: task completion 94.08%, time on task 89.16 seconds, abandonment rate 2.66%, and retry rate 0.25%, as shown in Table 5, demonstrating that objective engagement correlates with perceived usability.

The clustering of engagement scores into high-, medium-, and low-tier groups (Figure 10) reveals how caregivers interact with the EDT system. Users in the high-engagement cluster achieved strong task performance and high SUS scores, reflecting meaningful interactions. The medium engagement group showed satisfactory engagement with lower SUS scores, whereas the low-engagement group displayed limited interaction and lower usability perception. This analysis shows that users experience the system differently, highlighting the need to tailor system design and support for different engagement profiles. Demographic checks showed no significant difference in SUS scores by age (ANOVA *F*_4, 45_=0.399; *P*=.81, η²=0.034) or caregiving experience (ANOVA *F*_(3, 46_=1.58; *P*=.21, η²=0.093), indicating consistent perceived usability across the groups. The convergence of high perceived usability with high task performance provides evidence that the EDT system is usable and effective for caregivers. Higher SUS ratings correlated with more intensive interactions, demonstrating the system’s support for meaningful use in caregiving contexts. These behavioral indicators in the composite UES strengthen the predictive relationship between the UES and the SUS, showing that engaged caregivers achieve higher task success and perceive better usability.

### Theoretical Implications

The results demonstrate the complementarity between subjective and objective measures. While the SUS provides standardized user perception data with benchmarking values [19,22], it cannot reveal how users succeed or struggle during interactions [64]. By combining the SUS with UES behavioral analytics, our study showed that objective measures strengthen construct validity and highlight engagement heterogeneity that postuse scores may miss. In developing the UES, we refined the engagement score using foundational indicators of breadth, depth, and effectiveness of use, avoiding redundant measures, and ensuring interpretability. Equal weights were applied to the metrics, a defensible choice without prior evidence of differential importance. Future research could explore data-driven weighting strategies, such as principal component analysis, regression models, or SHAP analyses, using larger datasets [67].

Another important implication concerns the demographic sensitivity of the SUS. The ANOVA analysis showed no significant differences in usability scores by age or caregiving experience, suggesting that the SUS may be relatively stable across these demographic groups within this sample. The positive association between SUS and UES indicates that perceived usability and behavioral engagement are related. However, the present findings primarily demonstrate convergence between these measures rather than fully establishing their empirical complementarity. Future work should also examine more directly how both measures may capture distinct usability patterns and subgroup needs.

### Practical Implication for Health Care Systems

The findings of this study have important implications for the use of DT systems in health care settings. First, the alignment between high SUS scores and favorable task performance metrics suggests that designers should prioritize core workflows that caregivers value most: real-time vital sign monitoring, health trend visualization, abnormal event alerts, and AI-based recommendations. Maintaining simplicity in these functions is essential for building caregiver trust and encouraging adoption. Second, the clustering analysis of the composite UES showed varied system engagement levels, with a small subgroup in the low engagement cluster. This finding indicates the need for an adaptive user interface. Designers should provide configurable interaction modes, progressive onboarding, and contextual help features to address the diverse needs and digital literacy levels of caregivers. Such adaptations can reduce the engagement barriers among different user groups. Third, given the central role of predictive modeling in EDT systems, such as Bi-LSTM for heart rate prediction and long short-term memory for sleep stage interface, AI-generated insights must be transparent and actionable. Caregivers must distinguish between real-time data and predictions, with alerts that are accompanied by clear rationales. This enhances interpretability and reduces inappropriate decision-making in care settings.

Finally, this study emphasizes the need to support informal caregivers who frequently multitask and experience cognitive and emotional strain. EDT systems should adopt design strategies for rapid comprehension, including glanceable dashboards, consistent information hierarchies, and conservative notification policies to avoid alarm fatigue in users. This study demonstrates the value of behavioral analytics in pilot deployments, with system-generated logs supporting usability evaluation and providing feedback for refinement. Health care DT developers should integrate privacy-preserving analytics to monitor navigation issues and feature abandonment during real-world applications to guide design improvements and caregiver training. The results show that caregiver-facing DT systems must optimize core workflows while offering adaptability and transparency. These principles enable health care DT systems to support informal caregivers, enhance decision-making, and integrate digital health technologies into caregiving practices.

### Comparative Positioning and Methodological Contribution

To situate the usability of the EDT system within the broader digital health literature, we compared its SUS scores with those reported in prior studies on related technologies, as shown in Table 10. The EDT system achieved 80.45, which falls within the “excellent” range and exceeds the values reported for many comparable systems, such as VITAAL (58.3), Pocket Gait (59.7), and ED Health Information System (53.1). Some systems, such as the CHES eDiary (87.5), achieved similarly high SUS scores, although often in more narrowly defined use cases than the others. This benchmark highlights 2 important contributions to literature. First, the EDT system ranks among the higher-performing digital health technologies in terms of perceived usability, reinforcing its readiness for use in caregiver-facing applications. Second, unlike most prior studies that relied exclusively on the SUS or paired it with limited subjective feedback (eg, interviews and acceptability scales), our study integrated objective behavioral metrics derived from detailed user activity logs. This dual-method approach strengthens the robustness of valuation and provides a replicable methodological pathway for future assessments of DT technologies in health care.

**Table 10.** Comparative System Usability Scale scores of related studies and the elderly digital twin system.

Study/System	SUS^a^ score (reported)	Measures/Metrics used
HELMA [14]	72.2 (caregivers)	SUS + Partial usage logs
Engage [13]	66.3 → 82.6	SUS + NASA-TLX
VITAAL [15]	58.3	SUS + Feedback sessions
Pocket Gait [17]	59.7	SUS + Acceptability scale
CHES eDiary [45]	87.5	SUS + Walkthrough
TMS [16]	69.2	SUS
HeartAround [46]	62.2 → 78.8	SUS + Interview
ED Health Info System [47]	53.1	SUS + Interview
Our Study (EDT System)	80.45	SUS + Activity log metrics (UES^b^, tasks)

a SUS: System Usability Scale.

b UES: user engagement score.

### Limitations

Despite these strengths, several limitations must be acknowledged in this study. First, the sample size (n=50) limits the generalizability of the findings, and future research should validate the results with larger and more diverse caregiver populations. Second, the evaluation was conducted in a single-system context (the EDT prototype), which constrains external validity; usability outcomes may differ when deployed in varied care environments or integrated with other health systems. Third, although the SUS is a widely validated tool [63], it remains a subjective self-reported measure that can be influenced by user expectations, prior experience, or social desirability bias [68]. Although we mitigated this limitation by complementing SUS with objective behavioral logs, reliance on a single subjective scale may not fully capture all dimensions of usability. Finally, the cross-sectional study design limits our ability to assess the evaluation of engagement and usability perceptions evolved over time. Longitudinal studies are needed to capture the changes in caregiver interactions and satisfaction with sustained system use.

This study does not establish clinical validity or support the use of EDT as a diagnostic or treatment tool. The prototype was evaluated as a caregiver-oriented decision support and usability research system rather than as a regulated medical device, and the Fitbit Sense 2 used for sensing should be regarded as a consumer-grade wearable rather than a clinical reference device. In addition, the predictive components were evaluated only on the available study dataset, and no independent external sample validation was performed. Accordingly, the reported model performance metrics should be interpreted as preliminary indicators of technical feasibility rather than evidence of clinical-grade validity, external validity, or broader generalizability. Similarly, the LLM-generated outputs were intended to provide caregiver-friendly support, monitoring guidance, and follow-up suggestions rather than medical diagnosis or treatment advice. Furthermore, the composite engagement score was constructed using a pragmatic equal-weight additive approach, and formal testing of interdependence among its component metrics was not conducted.

### System Improvements and Future Work

This usability evaluation provided valuable lessons for refining the EDT. The system demonstrated excellent usability and high effectiveness, with a 94.08% task completion rate and low error rates. However, the findings reveal areas for enhancing system inclusivity and adoption. The small proportion of abandoned tasks (2.66%) and minimal retries (0.25 per task) indicated that certain interactions may challenge some users, particularly those in the low-engagement cluster identified in UES tiering. The average task time indicates overall efficiency but varies across engagement tiers. Medium- and low-engagement users required more time and retries, suggesting opportunities to streamline workflows. Simplifying navigation and reducing cognitive load can improve efficiency across different user profiles. Clustering analysis showed varied usability outcomes across users. Future development should address caregiver experience heterogeneity by tailoring features to different profiles, such as contextual prompts for low and advanced features for high-engagement users. Future work should expand the evaluation to more diverse caregiver populations, examine patterns of engagement and usability, incorporate contextual factors such as digital literacy and caregiving intensity, and evaluate the EDT in more naturalistic home care settings using scenarios grounded in real caregiver workflows to strengthen ecological validity. Future research should also examine intermetric correlations, dimensional structure, and alternative weighting strategies to further validate the composite engagement score. In addition, independent external datasets, prospective evaluation, and real-world deployment testing are needed to establish the robustness and generalizability of the predictive components more rigorously. Together, these efforts will help validate our findings and guide further improvements to ensure that the EDT system remains usable and responsive to caregiving practices.

### Conclusions

This study evaluated the usability of the EDT system by combining subjective perceptions with objective behavioral data. The findings demonstrated excellent usability and strong task performance, with high completion, low error, and abandonment rates. Correlation and regression analyses showed that the composite UES was not only positively associated with but also a significant predictor of usability, explaining 39.2% of the variance in SUS. Clustering further revealed differences in engagement tiers, underscoring that usability outcomes are not uniform across users. Taken together, these results highlight the value of integrating subjective and objective measures to capture a comprehensive picture of product usability. This study contributes methodologically by linking behavioral engagement metrics with perceived usability, particularly by identifying the strengths and areas for improvement in EDT systems. Future studies should validate these findings in larger and more diverse caregiver populations, explore longitudinal engagement patterns, and incorporate adaptive features to support users with varying engagement profiles.

## Supplementary material

10.2196/91873 Multimedia Appendix 1 Screenshots of the elderly digital twin system functions, interfaces, and key caregiver-facing modules.

## Abbreviations

AI: artificial intelligence
Bi-LSTM: bidirectional long short-term memory
CTR: click-to-task ratio
DT: digital twin
EDT: elderly digital twin
FU: feature usage
HRV: heart rate variability
ISO: International Organization for Standardization
LLM: large language model
NASA-TLX: National Aeronautics and Space Administration—Task Load Index
NF: navigation frequency
RR: retry rate
SpO₂: peripheral oxygen saturation
SUS: System Usability Scale
TAR: task abandonment rate
TC: task completion
ToT: time on task
UES: user engagement score
UMUX: usability metric for user experience
