# Supplementary material for: Combining Subjective Perceptions and Objective Behavioral Metrics With the Elderly Digital Twin System: Quantitative Usability Study
Source: JMIR Aging. 2026 May 26;9:e91873. doi: 10.2196/91873 (PMC13207158; doi:10.2196/91873)

Figure S1. Sleep metric analysis user interface.

The sleep metrics module displays sleep efficiency, sleep latency, number of awakenings, sleep transition patterns, and fragmentation metrics, with optional AI-assisted recommendation access when abnormal values are detected.

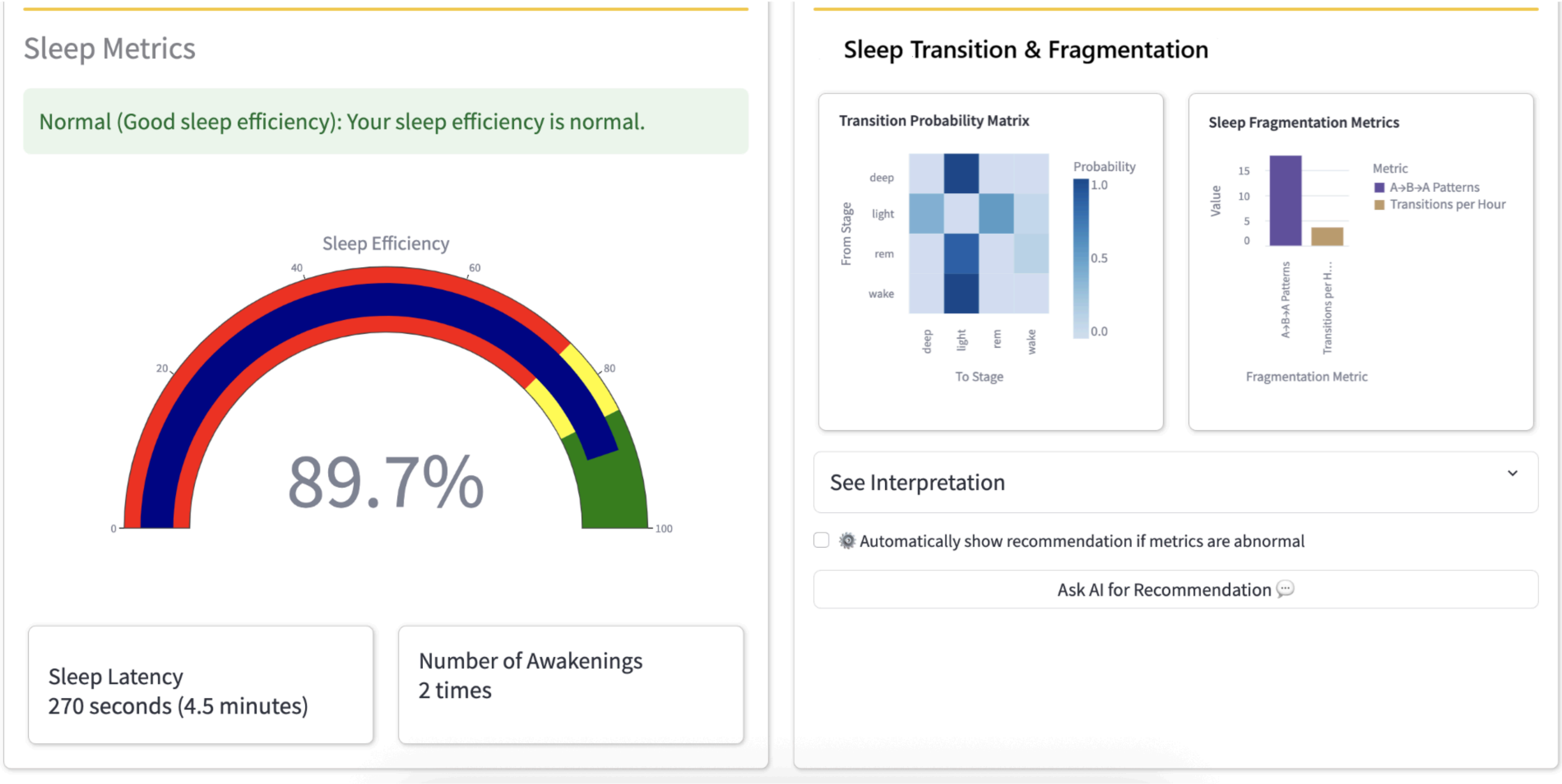

Figure S2. Sleep benchmark analysis user interface.

The sleep benchmark interface compares stage-level sleep distribution against expected ranges and presents an interpretation panel alongside AI-generated sleep recommendations.

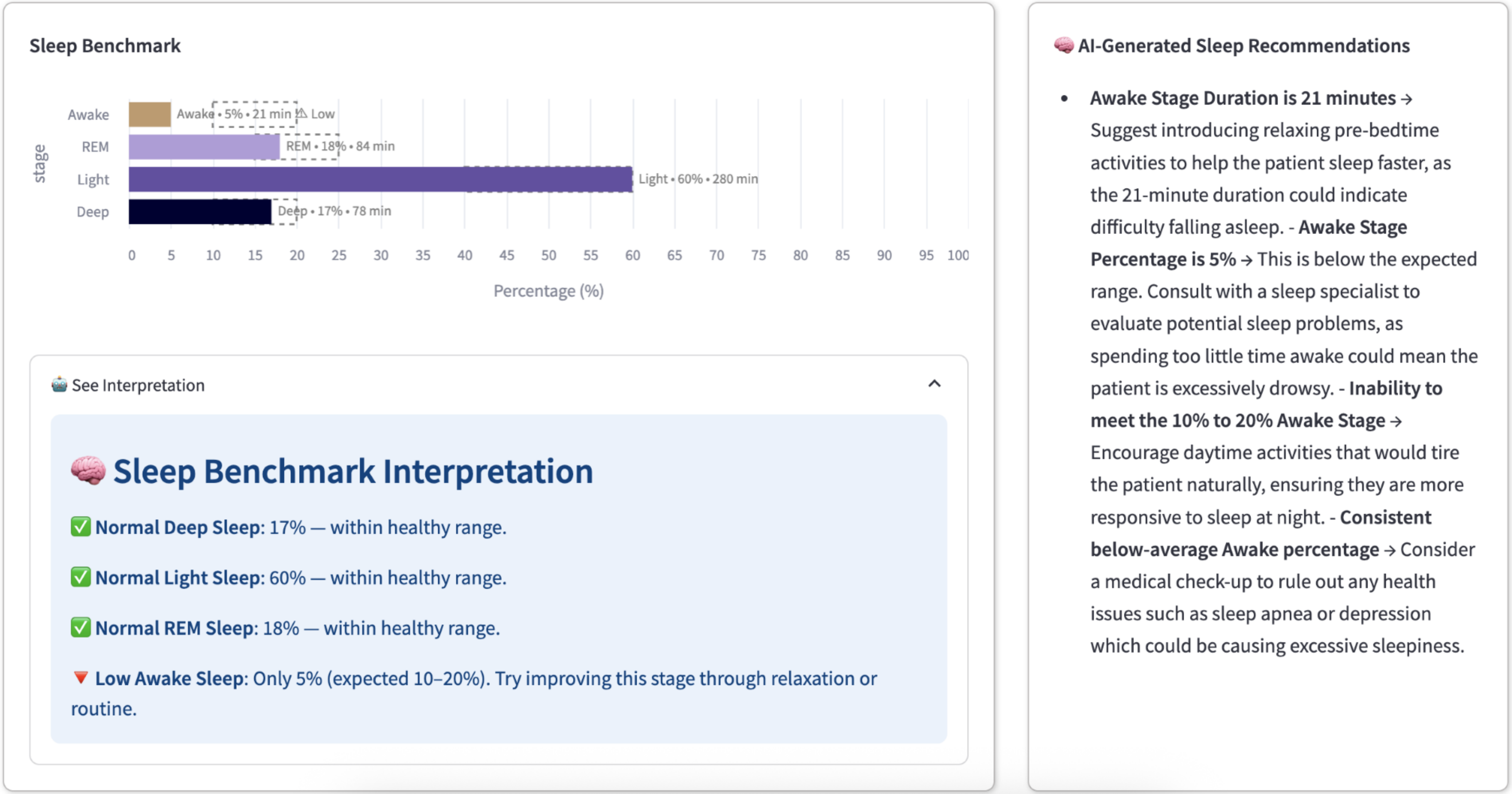

Figure S3. Blood oxygen saturation monitoring for hypoxemia detection model interface.

The SpO2 monitoring module visualizes overnight oxygen saturation trends, summarizes minimum, average, and maximum SpO2 values, and supports recommendation generation for hypoxemia-related follow-up.

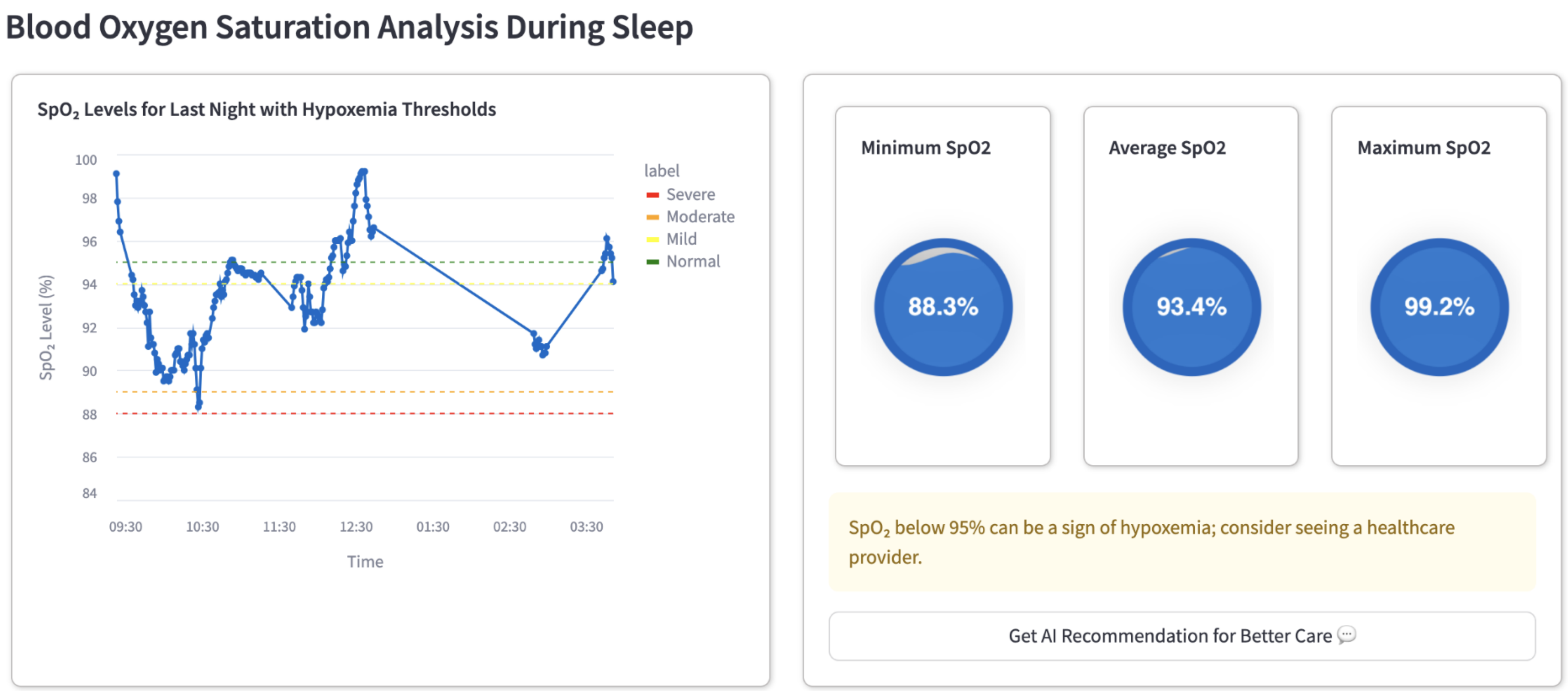

Figure S4. AI chatbot assistant interface.

The AI chatbot assistant enables caregivers to ask natural-language questions about physiological indicators and receive caregiver-friendly explanations and follow-up guidance.

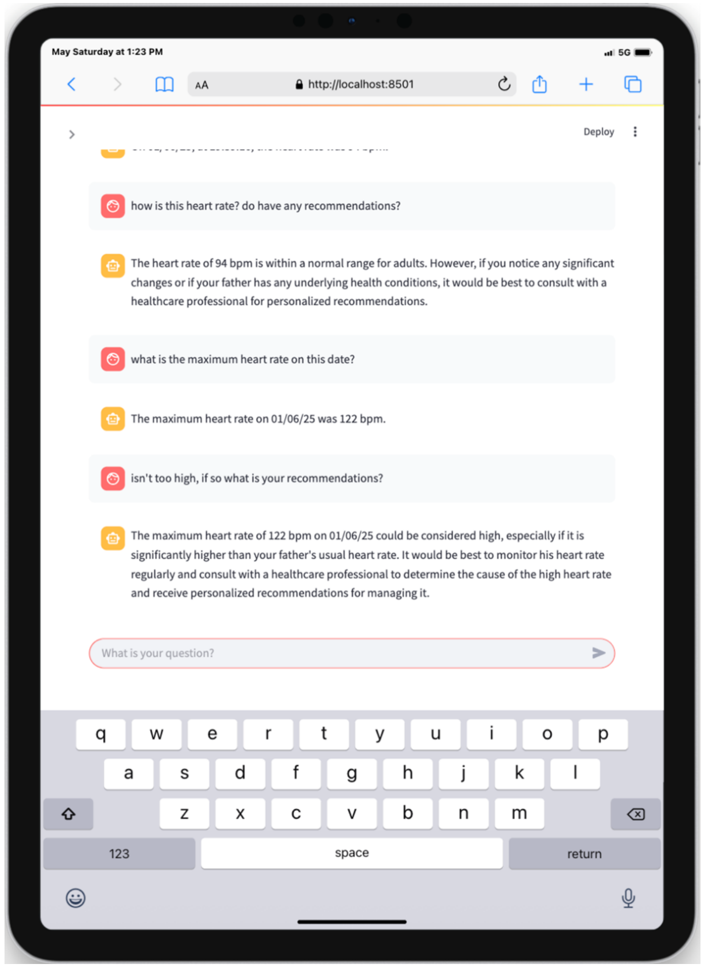

Supplement: Multimedia Appendix 1 [file aging-v9-e91873-s001.pdf]
